# Laser-Induced Apoptosis of Corticothalamic Neurons in Layer VI of Auditory Cortex Impact on Cortical Frequency Processing

**DOI:** 10.3389/fncir.2021.659280

**Published:** 2021-07-12

**Authors:** Katja Saldeitis, Marcus Jeschke, Eike Budinger, Frank W. Ohl, Max F. K. Happel

**Affiliations:** ^1^Department of Systems Physiology of Learning, Leibniz Institute for Neurobiology, Magdeburg, Germany; ^2^Auditory Neuroscience and Optogenetics Group, Cognitive Hearing in Primates Lab, German Primate Center, Göttingen, Germany; ^3^Institute for Auditory Neuroscience, University Medical Center Goettingen, Göttingen, Germany; ^4^Center for Behavioral Brain Sciences, Magdeburg, Germany; ^5^Institute of Biology (IBIO), University Magdeburg, Magdeburg, Germany; ^6^Medical School Berlin, Berlin, Germany

**Keywords:** auditory cortex, corticothalamic, laser-induced ablation, Mongolian gerbil (*Meriones unguiculatus*), current-source density, intracortical microstimulation

## Abstract

Corticofugal projections outnumber subcortical input projections by far. However, the specific role for signal processing of corticofugal feedback is still less well understood in comparisonto the feedforward projection. Here, we lesioned corticothalamic (CT) neurons in layers V and/or VI of the auditory cortex of Mongolian gerbils by laser-induced photolysis to investigate their contribution to cortical activation patterns. We have used laminar current-source density (CSD) recordings of tone-evoked responses and could show that, particularly, lesion of CT neurons in layer VI affected cortical frequency processing. Specifically, we found a decreased gain of best-frequency input in thalamocortical (TC)-recipient input layers that correlated with the relative lesion of layer VI neurons, but not layer V neurons. Using cortical silencing with the GABA_*a*_-agonist muscimol and layer-specific intracortical microstimulation (ICMS), we found that direct activation of infragranular layers recruited a local recurrent cortico-thalamo-cortical loop of synaptic input. This recurrent feedback was also only interrupted when lesioning layer VI neurons, but not cells in layer V. Our study thereby shows distinct roles of these two types of CT neurons suggesting a particular impact of CT feedback from layer VI to affect the local feedforward frequency processing in auditory cortex.

## Introduction

Being positioned at the nexus between the ascending subcortical and descending higher-cortical auditory pathway, the auditory cortex (ACx) is central for auditory processing and behavior (Scheich et al., 2007; Schreiner and Winer, 2007; Budinger et al., 2008; Sharpee et al., 2011; King et al., 2018). A prominent hypothesis is that sensory-related population activity in ACx is generated and dynamically adjusted through recurrent feedback processing with its thalamic relays via gating mechanisms (Suga, 1977; Zhang et al., 1997; Destexhe, 2000; Yu et al., 2004; Crandall et al., 2015; Guo et al., 2017). Corticothalamic (CT) feedback signals from ACx shape the receptive field and filtering properties of neurons in the auditory thalamus, the medial geniculate body (MGB; Suga and Ma, 2003; Zhang et al., 1997; Bäuerle et al., 2011; Anderson and Malmierca, 2013; Malmierca et al., 2015), and control the gain of thalamocortical (TC) transmission (Deschênes and Hu, 1990; He, 1997; Yu et al., 2004; Happel et al., 2014; Williamson and Polley, 2019). Such gain control via CT circuits was also shown for the visual (Olsen et al., 2012; Kirchgessner et al., 2020) and somatosensory system (Temereanca and Simons, 2004; Mease et al., 2014; Crandall et al., 2015).

CT neurons have been implicated in many cognitive functions, such as the regulation of attentional processes (Bollimunta et al., 2011), the perception of complex sounds (Homma et al., 2017), and dopamine-dependent auditory detection and discrimination learning (Happel et al., 2014; Guo et al., 2017; Deliano et al., 2018). CT feedback arising from cortical layers VI and V have distinct intra- and subcortical projection patterns to lemniscal and non-lemniscal thalamic nuclei (Hazama et al., 2004; Rouiller and Durif, 2004; Llano and Sherman, 2009). Recently, Williamson and Polley (2019) have suggested that the broader corticofugal projection neurons in layer V broadcast sensory inputs to distributed downstream targets, while CT neurons in layer VI regulate specifically TC response gain and selectivity. Layer VI neurons thereby exert strong feedforward amplification at the level of local columnar circuits adaptively during different behavioral states (Augustinaite and Kuhn, 2020; Clayton et al., 2020).

In a previous study, ferrets whose layer VI CT neurons of the ACx had been selectively lesioned by means of a chromophore-targeted laser photolysis showed impaired perceptual grouping of harmonics—one of the key cues in the perception of complex sounds (Homma et al., 2017).

Here, we applied this method in the Mongolian gerbil (*Meriones unguiculatus*) to investigate the contributions of CT neurons to acoustically and electrically evoked population activity patterns of primary ACx (field AI) as revealed by current-source density (CSD) analysis (Happel et al., 2010). Targeting of CT neuronal somata was achieved by injecting laser-activatable cytolytic chromophores attached to retrograde beads (Macklis, 1993; Bajo et al., 2010) into the MGB.

While the canonical spatiotemporal CSD pattern across cortical laminae evoked by acoustic stimulation (AcS) was generally preserved, photolytic apoptosis of specifically layer VI neurons led to frequency-selective changes with respect to strength and timing of columnar current flow. We found a reduced contrast between responses evoked by the best frequency (BF) and frequencies 2 octaves away of the BF (non-BF) in the main thalamorecipient layer IV (early granular sink, S1) and reduced BF-evoked input in layers Vb/VI (early infragranular sink, iS1). In contrast, current flow in layers I/II was increased (late supragranular sink, S2).

Infragranular intracortical microstimulation (ICMS) in ACx evoked local-field responses (Deliano et al., 2009) and translaminar CSD patterns (Happel et al., 2014) similar to acoustic stimulation. Here, we could demonstrate that intact CT feedback from layer VI is crucial for this electrically evoked columnar population pattern: Selective apoptosis of CT neurons in layer VI diminished this direct ICMS-evoked columnar activation significantly. This finding confirms our hypothesis that infragranular microstimulation activates a fast-acting recurrent CT loop via the ventral part of the MBG (MGv; Happel et al., 2014).

Our study thereby shows that particularly layer VI CT neurons affect the columnar frequency processing in ACx through a frequency-specific gain in TC-recipient layers.

## Materials and Methods

### Experimental Animals

Experiments were performed on 21 adult male ketamine-xylazine anesthetized Mongolian gerbils (age: 4–6 months, body weight: 65–85 g). All experiments were conducted in accordance with the international NIH Guidelines for Animals in Research and with ethical standards for the care and use of animals in research defined by the German Law for the protection of experimental animals. Experiments were approved by an ethics committee of the state Saxony-Anhalt, Germany.

### Experimental Design

**FIGURE 1:**
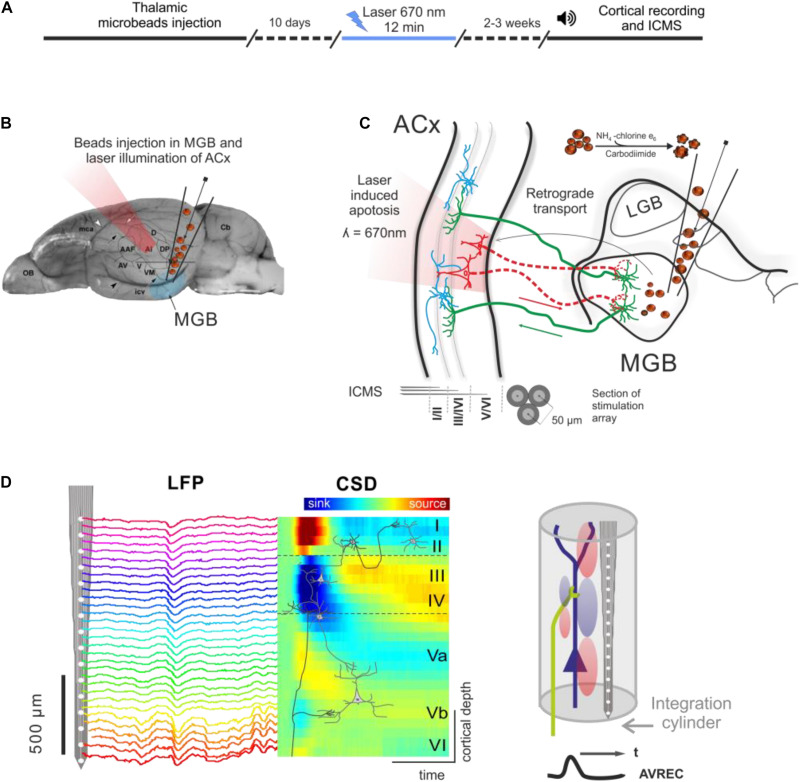
Experimental design and photolytic apoptosis technique. **(A)** Timeline of the experiments from thalamic microbeads injection, laser illumination of ACx (10 days later), and cortical recordings (2–3 weeks later). **(B)** Unilateral injection of conjugated red retrobeads with chlorin e6 into the MGB is followed by transcranial illumination of the ACx 10 days later. **(C)** Retrograde transport to the CT projection neurons and laser illumination induces a photolytic apoptosis of CT feedback by the release of reactive oxygen species. Electrodes used for intracortical microstimulation (ICMS) were placed in supragranular, granular and infragranular layers of the auditory cortex. **(D)** Laminar recordings of the local field potential (LFP) across all cortical layers in the ACx are transformed into the current source density (CSD) distribution in order to map the spatiotemporal profile of synaptic transmission. Current sinks are thereby interpreted as locations of excitatory synaptic input (*right*). Roman numbers indicate cortical layers, as in all following figures. Dashed lines indicate boundaries between supragranular, granular and infragranular layers.

**FIGURE 2:**
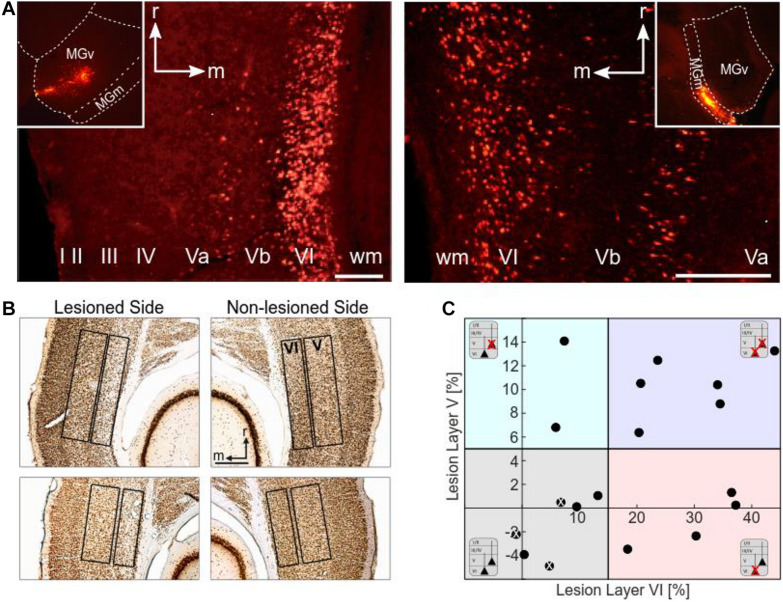
Histological analysis. **(A)** Retrograde labeling of CT neurons. Images by fluorescence microscopy show examples of injection sites into the MGv (left inset) and MGm (right inset) of the MGB, and the corresponding retrograde labeling in layers VI and V of the ACx (field AI). MGv receives cortical input mainly from layer VI, but also from layer V. Cortical neurons from layers VI and V project to MGm. Scale bars: 200 μm (A left), 100 μm (A right). **(B)** Histological analysis of neuronal loss of the animals used for CSD analysis. Top: NeuN-stained section showing neuronal apoptosis in layer VI of the ipsilateral, i.e., illuminated cortical side of AI (case G12). Cellular density is much lower than in the contralateral non-lesioned side. Bottom: NeuN-stained section showing neuronal apoptosis in layers V and VI of the illuminated cortical hemisphere in another case (G13). Lesions in layer V were not as strong and not as clearly visible as layer VI lesions. Scale bar: 500 μm. **(C)** Scatter plot representing the percentage neuronal loss (positive values) compared to the contralateral side in layers V and VI of the individual animals (negative values result from statistical variance around mean). There is no correlation between lesion strengths in layers V and VI (*r* = 0.394, *p* = 0.106). The different background colors represent the ranges of the four lesion groups to which the cases were assigned according to their lesion strengths in layers V and VI. Animals injected with saline instead of the tracer are labeled with a white x.

An outline of the experimental procedure is depicted in Figure 1. The photolytic apoptosis of chromophore-targeted neuronal populations has been performed before in different species including mice, rats, and ferrets (Macklis, 1993; Bajo et al., 2010; Homma et al., 2017). In this study, animals received stereotactic unilateral injections of the photolytic tracer into the MGB (Figures 1B,C). After retrograde transport to the ACx and fluorescent labeling of CT projection neurons (Figure 1C; see also Figure 2A), the ipsilateral ACx was illuminated with laser light (670 nm, 10 days after injection), which induces a photolytic apoptosis of CT projection neurons by the release of reactive oxygen species. Following completion of the apoptotic process, electrophysiological cortical population activity was recorded (Figure 1D). Afterward, neuronal cell loss can be made visible by immunohistochemical markers such as NeuN, SMI-32 neurofilament, and caspase 3 (Figure 2B and Supplementary Figure 1).

### Preparation of the Photolytic Tracer (Chlorin e6-Conjugated Retrobeads)

A 1 mM solution of Chlorin e6(-monoethylenediamineamide) (Phyto-chlorin, Frontier Scientific, United States, CAS# 19660-77-6, MW 596.68) was made up with 3 ml of 0.01 M PB (pH 7.4) and was activated with 5 mg N-Cyclohexyl-N’-(2-morpholinoethyl) carbodi-imide metho-p-toluenesulfonate (Sigma-Aldrich, Switzerland, CAS# 2491-17-0) for 30 min at 4°C on a rocker table (70 rpm). 50 μl Red Retrobeads IX (Lumafluor, United States, excitation: 530 nm, emission: 590 nm) were diluted in 300 μl PB and added to the solution. Chlorin e6 was then attached to the latex surface of the fluorescent microbeads by gentle agitation on a rocker table at 4°C. The reaction was stopped after 60 min with 335 μl 0.1 M glycine buffer (pH 8.0) and this mixture was pelleted by a series of high-speed centrifugations (Optima MAX Ultracentrifuge, Beckman Coulter, United States, 60 min each, 140,000 g, 45,000 rpm; MLA-80 rotor, Beckman Coulter; 10 ml Centrifuge Tubes, Beckman) until the supernatant was fully clear (about 4 times). Following each round, the supernatant was removed and the pellet resuspended in 3 ml PB. The final pellet was resuspended in 50 μl PB and stored at 4°C. Conjugated beads were injected within 14 days (Macklis, 1993).

### Tracer Injection Into the MGB

Immediately before use, the tube containing tracer solution was put into an ultrasound bath (Sonorex Super 10P, Bandelin, Germany, 15 min) to prevent clotting. Glass pipettes (outer diameter 1.2 mm, inner diameter 0.68 mm, WPI, United States; tip diameter broken to 20 μm) were filled backward using a 28 gauge MicroFil needle (WPI, United States). For the stereotaxic pressure injections, general initial anesthesia was induced intraperitoneally with a combination of 45% ketamine (10 mg/100 g body weight, Ratiopharm GmbH, Germany) and 5% xylazine (0.5 mg/100 g body weight; Rompun, 2%, Bayer, Germany) prepared in isotonic sodium chloride solution (50%). The level of anesthesia was controlled by monitoring the hindlimb withdrawal reflex and respiratory rate and maintenance doses were given as needed (~0.06 ml/h). Body temperature was kept at 37°C using a heating blanket. The cranial skin was disinfected, locally anesthetized, and incised. A small hole was drilled unilaterally with a dental drill into the skull according to the stereotaxic coordinates of the MGB established previously (Saldeitis et al., 2014; Radtke-Schuller et al., 2016; 3.9–4.0 mm caudal and 2.85–2.9 mm lateral from Bregma) and conjugated microbeads (40 nl) were injected over a period of 2 min. The injection was performed with the help of a fine glass micropipette (outer diameter 1.2 mm, inner diameter 0.68 mm, WPI, United States), which was pulled (Sutter Instruments, United States), broken (tip diameter: 20 μm), and then mounted on an oil hydraulic nanoliter delivery system (WPI). The micropipette was advanced vertically into the brain. The depth of the tip, measured from the cortical surface, was 4.1–4.5 mm. Following the injections, the cranial opening was closed with bone wax (Ethicon, Germany), the surgical site was treated with an anti-inflammatory ointment (Volon A, Dermapharm GmbH, Germany), and the skin over the cranial opening was closed with a tissue adhesive (Histoacryl, Braun, Germany).

### Laser Illumination of AI

Ten days following the injection, photolytic apoptosis of retrogradely labeled cortical neurons was induced by ipsilateral exposure of AI to laser light. Under anesthesia with ketamine (10 mg/100 g body weight) and xylazine (0.5 mg/100 g body weight) the skin and the temporal muscle overlaying the ACx were deflected laterally. The exposed AI, which can be identified by its vasculature landmarks (e.g., Thomas et al., 1993; Sugimoto et al., 1997) was illuminated transcranially with a 670-nm wavelength near-infrared light from a tunable 300 mW laser diode (Flatbeam-Laser 670, Schäfter + Kirchhoff, Germany). The laser light was adjusted with beam-shaping optics to create a 1.35-mm spot focused at the level of layer V/VI (1–1.5 mm deep) and the laser intensity was tuned to 50 mW (surface energy doses of ~1,250 J/cm^2^, exposure area approx. 2.86 mm^2^) and maintained for 10–12 min (5–6 min at two cortical sites). Following illumination, the skin was closed using surgical thread and tissue adhesive (Histoacryl, Braun, Germany), and the animal was allowed to recover.

### Surgery and Electrophysiological Recordings

Surgical and experimental procedures have been described in detail previously (Happel et al., 2014; Saldeitis et al., 2014; Brunk et al., 2019).

After 2–3 weeks post-laser exposure, gerbils were anesthetized and monitored as described above. The ipsilateral ACx was exposed by craniotomy (≈3 × 4 mm) of the temporal bone. Recordings were performed in an acoustically and electrically shielded recording chamber. Laminar profiles of local field potentials (LFP) were measured using linear 32-channel-shaft electrodes (NeuroNexus, 50 μm inter-channel spacing, 413 μm^2^ site area; type A1x32-5mm-50-413) inserted perpendicular to the cortical surface. Neuronal potentials were pre-amplified (500x), band-pass filtered between 0.7 and 170 Hz (3 dB cut-off frequency), digitized at 2 kHz (Multichannel Acquisition Processor, Plexon Inc.) and averaged over 40–80 stimulus repetitions. The location of the field AI in primary ACx was identified by vasculature landmarks and physiological parameters (Ohl et al., 2000, 2001; Brunk et al., 2019; Deane et al., 2020).

### Auditory Stimulation and Estimation of Cortical Tuning

We presented pseudo-randomized series of pure tones (duration: 100 ms with 5 ms sinusoidal rising and falling ramps; inter-stimulus interval: 600 ms; digitally synthesized using Matlab and converted to analog signals by a data acquisition National Instruments card; PCI-6711) spanning eight octaves from 250 Hz to 32 kHz and using different sound pressure levels (34–74 dB SPL). Sound pressure intensities were calibrated prior to the experiments by means of a reference signal (0 dB attenuation corresponds to 94 dB SPL). Stimuli were delivered via a programmable attenuator (g.PAH, Guger Technologies; Austria), a wide-range audio amplifier (Thomas Tech Amp75) and a loudspeaker (Tannoy arena satellite KI-8710-32) positioned at 1 m distance in front of the animal’s head. The response threshold was determined as the lowest intensity eliciting a significant response at any frequency 3SD over baseline.

### Layer-Specific Intracortical Microstimulation

Intracortical microstimulation of biphasic (current-balanced), monopolar, cathodic-first rectangular single pulses were applied (phase duration: 100 μs, inter phase interval 50 μs, ISI: 500 ms, repetitions: 50) in three different cortical depths corresponding to supragranular, granular, and infragranular layers (SGstim, Gstim, IGstim). Stimulation arrays consisted of three attached Teflon-insulated stainless steel wires (Ø with isolation 50 μm; California Fine Wire) implanted at cortical depths of 100, 600, and 1,200 μm, respectively (see Figure 1C). The array was inserted proximal (300–400 μm ventrally, i.e., into the proposed same isofrequency contour) to the recording electrodes. Stimulation amplitudes were varied from 40 to 160 μA. Electrical stimuli were generated with a PC and a programmable electrostimulation device (STG2008, Multichannel Systems, Germany). The shape of the stimuli was generated using Matlab and sent to the stimulus generator.

### Pharmacological Silencing of Cortical Activity

After recording of acoustically and electrically evoked CSD patterns of pharmacologically untreated animals, the GABA_*A*_-agonist muscimol (7.5–8.4 mM, 20–30 μl, Tocris, United States; dissolved in 0.9% sodium-chloride) was applied onto the cortical surface for pharmacological blocking of intracortical transmission (Edeline et al., 2002). Axonal conductance should not be influenced; electrical stimulation of the cortex should therefore be able to excite for example CT projection fibers. Inputs with their neuronal generators outside of the pharmacologically inhibited region, like TC projections, should also still be excitable. The volume and concentration of muscimol used in this study has been shown to be an appropriate dosage for effective cortical silencing in gerbil ACx (Happel et al., 2010; Happel and Ohl, 2017). During diffusion of muscimol, acoustic stimuli (pure tones at 40 dB attenuation) were presented to monitor which layers have been silenced so far. After complete diffusion of muscimol across all cortical layers (takes approximately 0.5–1 h; see also Happel et al., 2010) the same set of acoustic and electrical stimuli was repeated.

### Current Source Density Analysis

One-dimensional CSD profiles were calculated from the second spatial derivative of the LFP (Mitzdorf, 1985; Schroeder et al., 1998):

$$∼CSD\approx \frac{\delta^{2}\theta \left(z\right)}{\delta z^{2}}=\frac{\theta \left(z+n△z\right)-2\theta \left(z\right)+\theta \left(z-n△z\right)}{{\left(n△z\right)}^{2}}$$

where θ is the field potential, z the spatial coordinate perpendicular to the cortical laminae, Δ*z* the spatial sampling interval (50 μm), and *n* the differentiation grid. LFP profiles were smoothed with a weighted average (Hamming window) of 9 channels (corresponding to a spatial filter kernel of 450 μm; linear extrapolation of 4 channels at boundaries; see Happel et al., 2010). Main sink components were found to represent the architecture of primary sensory input from medial geniculate body (MGB). The sink that is associated with the main projections from the ventral division of the MGB (MGv) onto pyramidal neurons in cortical layers III/IV is referred to as S1. Collaterals of these TC projections also target infragranular layer Vb/VI (Winer et al., 2005; Constantinople and Bruno, 2013; Saldeitis et al., 2014; Schaefer et al., 2015), which result in the so-called early infragranular sink (iS1). Later CSD components include the supragranular sink S2 (layers I/II), and the infragranular sink S3 (layer Va).

In order to compare responses evoked by different stimuli (acoustic, electrical) and during treatments (pre/post muscimol) conditions, channels assigned to a corresponding cortical layer were kept constant. The location of electrically evoked S1 was derived from the location of the acoustically evoked granular sink. To facilitate comparison of activation between animals and/or conditions, we decided to always use the same time window for analysis of a given CSD sink, in which the respective sink could occur according to its definition (AcS: S1: 10–50 ms, iS1: 10–40 ms, S2: 40–300 ms, S3: 40–300 ms; ICMS: S1: 6–20 ms, iS1: 6–20 ms). We have chosen 10 and 6 ms as lower boundaries for AcS and ICMS, due to minimal onset latencies and length of stimulation artifacts, respectively.

For each animal and stimulus condition, we determined the tone-evoked mean integrals [INT; in (mV/mm^2*^ms)] calculated for all identified acoustically evoked sinks at 54 dB SPL by averaging across the corresponding CSD channels for the above given time windows. Importantly, we only considered negative (i.e., sink), but not positive (i.e., source) components of the INT to prevent distortions related to individually different sink durations. We further analyzed onset latencies (OL) of all acoustically evoked sink components, i.e., the time point, at which the response threshold was first exceeded for at least 5 ms. We determined a best frequency (BF) as the frequency of the stimulus set that elicited the highest amplitude/integral of the initial granular sink S1. The sharpness of frequency tuning was explored by calculating the change of the response two octaves away from the BF (non-BF) relative to the BF-evoked response in percent.

$$\Delta Resp.nonBF\left[\%\right]=\frac{Resp.nonBF-Resp.BF}{Resp.BF}*100$$

For latency analysis, the differences between the respective onset latencies were analyzed. Since the late extragranular sinks (S2, S3) depend on initial activation, their values were related to the S1 measures.

We also transformed the CSD by rectifying and averaging waveforms of each channel (n) comprising the laminar CSD profile (AVREC).

$$AVREC=\frac{\sum_{i=1}^{n}\left|CSD_{i}\right|\left(t\right)}{n}$$

While information on the direction and laminar location of transmembrane current flow is lost by rectification, the AVREC waveform provides a useful measure of the temporal pattern of the overall strength of transmembrane current flow (Schroeder et al., 1998).

To compare the overall activation strength between animals, AVREC integrals within time windows from 10 to 50 ms (acoustic stimulation) and from 6 to 50 ms (ICMS) were calculated.

For comparison of different lesion groups (non/weakly lesioned, layer VI lesioned, layer VI plus layer V lesioned, see Figure 2C), to which the individual cases were allocated according to the histological results (see below), CSD profiles of animals belonging to the same group were spatially aligned with respect to the granular sink S1 and then averaged (Szymanski et al., 2009). Similarly, AVREC curves obtained from acoustic and electrical stimulation were averaged and plotted including standard error.

### Immunohistochemistry

Following the electro-physiological experiments, the gerbils were perfused transcardially with 20 ml of 0.1 M PBS (pH 7.4) followed by 4% PFA (200 ml). Brains were postfixated in 4% PFA overnight, cryoprotected in 30% sucrose dissolved in PBS, frozen and cut into 50 μm thick horizontal slices. Every first out of three sections was mounted on slides and coverslipped using Immu-Mount (Thermo Scientific, Germany) to analyze the injection site and retrograde transport of beads under a fluorescence microscope. In addition, every second out of three sections was stained to visualize neuronal nuclei (NeuN) to verify the efficacy of the laser treatment indicated by reduction of cell number in layers V and VI. To this aim, sections were incubated in a solution containing a monoclonal mouse antibody to NeuN (1:1,000, Chemicon Europe), 0.1–0.3% Triton, and 1% BSA for 2 days. The brains of three pilot animals, which served to validate the photolytic apoptosis method, were in addition processed for the neurofilament protein SMI-32 (monoclonal mouse IgG, 1:5,000) and Caspase 3 (polyclonal rabbit IgG, 1:2,000), a key mediator of apoptosis. To ensure specificity of the later secondary antibody, control probes without primary antibodies were also made. After blocking against unspecific binding sites, appropriate secondary biotinylated antibodies were used (anti-host IgG 1:200, Vector Labs). The reaction product was visualized by incubating the sections in the ABC-solution (Vectastain Elite ABC Kit, Vector Labs) and using 3,3’-diaminobenzidine (0.4 mM of DAB, Sigma-Aldrich) as chromogen in the presence of 0.015% H_2_O_2_. After rinsing with TRIS-HCl (1x) and PBS (2x), the sections were mounted on gelatine-coated slides. The sections were dehydrated in isopropanol (2 min) and Roticlear (ROTH, Germany, 3 × 5 min) and then coverslipped using Merckoglass (Merck, Germany).

### Histological Analysis

Light and fluorescence microscopic analyses and photography (to verify injection sites and determine lesion efficacy) were carried out using a microscope (Zeiss Axioskop 2, Germany), fitted with the appropriate filters for fluorescence and a digital camera (Leica DFC 500, Germany).

Calculations of neuronal cell loss in order to evaluate possible effects of neuronal lesions on cortical activation were made using ImageJ^1^. To this aim, color photographs of NeuN-stained sections were converted to 16 bit images (gray scale), and then to binary (black/white) images by choosing a threshold that removes as much background as possible without removing cells. The same threshold was applied for all sections of a given animal. Layers V and VI, which are well discernible in NeuN stained tissues, were outlined in six sections on average per hemisphere surrounding the electrodes on the lesioned side and, for comparison, at similar dorsoventral levels of the contraleral AI.

Then, as a measure of cell density, the percentage areas occupied by black particles (i.e., NeuN stained nuclei) within these laminar contours were determined, which served to calculate the percentage neuronal loss in both infragranular layers of the illuminated side relative to the contralateral side for each animal.

### Statistical Analysis

To reveal possible gradual effects of CT lesions (see Figures 4, 6), we performed simple linear correlation analysis between the measured values and lesion strength in either layer V or VI (two-sided, significance level: 0.05). To account for both layer V and VI effects simultaneously, we fitted a linear mixed effects model by means of the Matlabs fitlme function [Maximum likelihood estimation; t-statistics (for testing the null hypothesis that the coefficient is equal to zero)] using residual degrees of freedom with layer V and layer VI lesions as fixed, and subject as random factors. The predictor variables were treated as either continuous (exact individual cell losses) or categorical (grouped; i.e., considered as either lesioned or non-lesioned according to lesion strength thresholds). We thereby fitted pre- and post-muscimol data as dependent variable (Dep.Var) separately (see Supplementary Tables 1, 2).

Dep.Var∼LesV+LesVI+(1|Subject)

To test for potential statistical interaction between layers, a model including an interaction term (LesV^∗^LesVI) was also constructed. As all but one comparison were found to be insignificant, we opted for the simpler model without an interaction term resulting in increased statistical power. For graphical purposes, animal groups were pooled (animals with vs. animals without layer VI lesions, independent of lesion strength in layer V and vice versa), where applicable.

The effect of pharmacological cortical silencing and its possible interaction with CT cell loss was analyzed using a different mixed effects model:

$$\begin{array}{c} Dep.Var∼LesV*Treatment+LesVI*Treatment\\ +\left(1|Subject\right) \end{array}$$

where Dep.Var contains both pre and post muscimol measurements (see Supplementary Table 3).

## Results

After photolytic lesioning of auditory CT projection neurons in Mongolian gerbils we performed *in vivo* multichannel recordings of LFP and laminar CSD distributions from primary ACx (field AI) to investigate the impact of the lesions on auditory cortical processing at the circuit level using acoustic and electrical stimulation. This was done before and after cortical silencing with muscimol to dissociate the TC from polysynaptic intracortical inputs.

### Method Validation

The areal and laminar specificity of the retrograde transport and photolytic apoptosis was evaluated by complementary microscopic inspection of fluorescent (i.e., location of retrobeads) and NeuN- and SMI32-stained brain sections (Supplementary Figures 1A,B, 2) of three pilot animals. Casp3-stain was used to confirm the completion of the apoptotic process at the selected time after laser illumination (Supplementary Figure 1C). Responsiveness of thalamic neurons near the injection site of the photolytic tracer was also checked in these animals (for details, see Supplementary Figure 3). The CSD profiles found in the six animals of our study without or with fairly weak CT cell loss (“non-lesioned” group), which were likewise exposed to the laser treatment, closely matched with those from untreated animals based on previous data (Happel et al., 2010; Happel and Ohl, 2017; Deane et al., 2020). We use this group of animals to demonstrate that the laser illumination did not unspecifically interfere with cortical physiology.

### Laminar Origin of the Auditory CT Connections in Mongolian Gerbils

Retrogradely labeled CT neurons were located in layers VI and V. The number of fluorescent beads in layer VI of primary field AI of the ACx was particularly high after injections into MGv (Figure 2A, left). Labeling or apoptosis in layer V of AI and the anterior auditory field AAF was stronger if terminals in the dorsal (MGd) or medial division of the MGB (MGm) had incorporated the tracer (Figure 2A, right). In the posterior fields, prominent labeling in layer V was found after tracer deposits into any of the MGB divisions. Due to the wide intracellular distribution of the beads, many cells could be identified as pyramidal neurons by their characteristic shape including their basal and apical dendrites. The topography of projections from AI to MGv was tonotopic but not with the same anatomical precision compared to the TC projections (Saldeitis et al., 2014). This led to considerable labeling across the ACx even with confined thalamic injections.

### Histological Quantification of Laser-Induced Neuronal Loss

**FIGURE 3:**
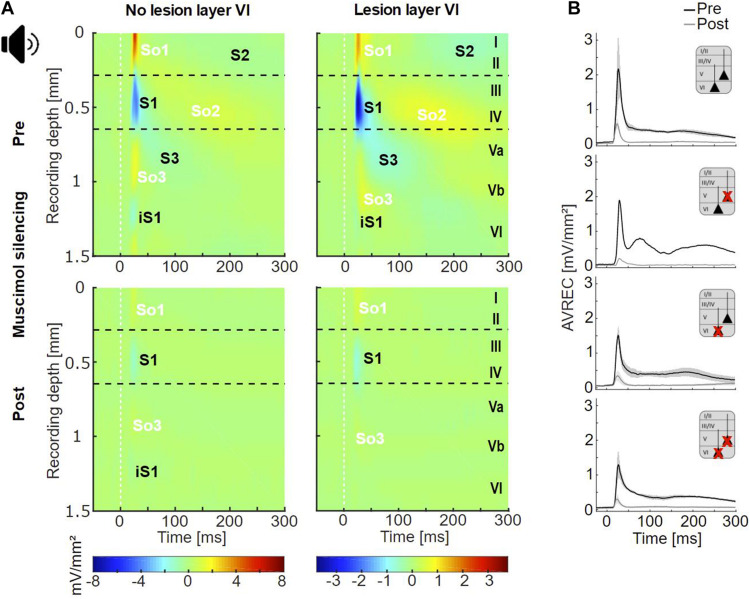
Cortical responses evoked by acoustic stimulation. **(A)** Averaged acoustically evoked CSD profiles at BF (54 dB SPL), before (top), and after (bottom) cortical silencing with muscimol. The grand mean of cases without lesions in layer VI (lesion groups non-Les and LesV, *n* = 7) and that of cases with layer VI lesions [groups LesVI and LesV+VI, *n* = 10 (pre), 9 (post)] had comparable general activation patterns of current sinks (S1, S2, S3, iS1) and sources (So1, So2, So3), shown in blue and yellow/red, respectively. Animals without layer VI lesions displayed a stronger early infragranular sink (iS1). **(B)** Averaged AVREC traces at BF before (black) and after (gray) cortical silencing showing means (solid lines) and SEM (shaded areas) of the four lesion groups. Curves were similar across all groups. n_*non*__–__Les_ = 6, n_LesV_ = 1, n_LesVI_ = 4; n_LesV__+__*VI*_ = 6 (pre), 5 (post).

To assess potential relationships between lesion strengths of CT connections and their possible physiological consequences we verified the thalamic injection sites (see Table 1) and quantified neuronal cell loss in the infragranular layers of AI. Images of brain slices stained for NeuN were used to determine the lesion efficacy, the contralateral side serving as reference. Moderate to strong neuronal loss, as frequently present in layer VI, was clearly visible (Figure 2B), whereas the rather weak lesions in layer V (Figure 2B, bottom) were less obvious. Neuronal cell loss ranged up to ∼15% in layer V, and up to ∼40% in layer VI, which corresponds to ∼80% CT neurons of layer VI (Kelly and Wong, 1981; Prieto and Winer, 1999). Based on individual lesion efficacy (i.e., percent cell loss compared to the contralateral side), we assigned animals to different lesion groups; non- or weakly lesioned (non-Les), layer V lesioned (LesV), layer VI lesioned (LesVI), layer V plus layer VI lesioned (LesV+VI) (Figure 2C). The thresholds (5% loss for lesion in layer V, 15% loss for lesion in layer VI) were chosen based on the range of the side differences in the saline animals (up to 4.9% for layer V, and 6.8% for layer VI), the absolute range of lesion (14% vs. 44%) as well as on the effects of lesion on cortical physiology, although for most parameters, a gradual lesion effect was found. Main findings were not affected by shifting the threshold (e.g., of layer VI lesions to 10% (Supplementary Table 2B) or 20% (Supplementary Table 2C). The lesion strengths in layer V and VI did not correlate with each other (Figure 3C), reflecting the target-specific anatomy of CT connections and allowing separate statistical analyses. Further, including layer interaction terms in our statistical analysis (see “Materials and Methods”) did not result in significant alterations of main conclusions.

**TABLE 1:**
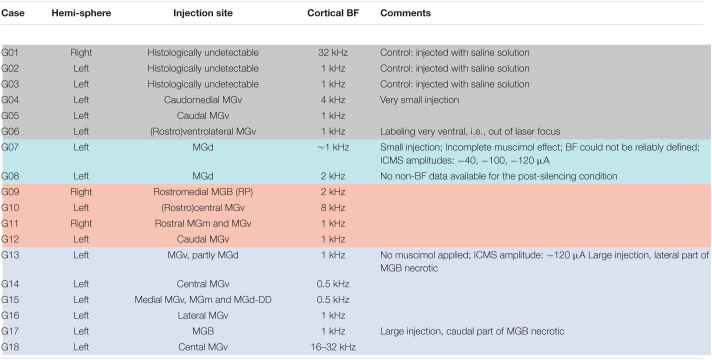
Experimental animals used for CSD analysis^*a*^.

*^a^For each case, the injected and illuminated hemisphere, intrathalamic location of the injection site, and the BF encountered at cortical recording site are depicted, and, where required, comments. Background colors indicate the respective group assignments defined by the lesion strengths, namely non-Les, LesV, LesVI, LesV+VI (see also Figure 2C). Control animals were injected into MGB with saline instead of the tracer solution.*

### Acoustically Evoked Columnar Processing

**FIGURE 4:**
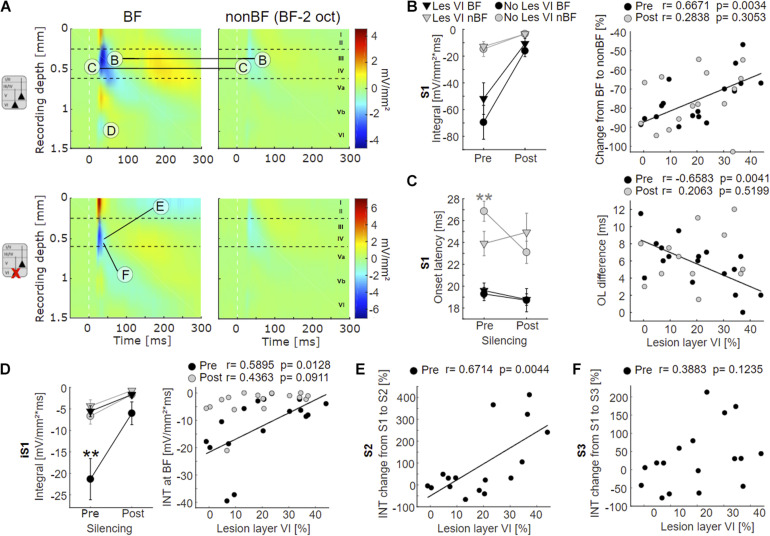
Effects of layer VI lesions on acoustically evoked cortical activity. **(A)** On the left, exemplary CSD profiles of a non-lesioned (case G04) and a layer VI lesioned (case G10) animal are represented, both at BF and at non-BF (2 octaves below BF). The letters refer to specific parameters and spatial locations used for the quantitative analyses shown in 4B-F. **(B)** Left: Means ± SEM of S1 integrals (INT), before and after application of muscimol at BF (black) and non-BF (gray), comparing animals without lesions in layer VI (circle) and those with lesions in layer VI (triangle). There is a trend toward stronger activation at BF in non-lesioned animals. Right: Relationships between lesion strength in layer VI and percentual change of non-BF INT, before (black circles) and after (gray circles) cortical silencing. In the pharmacologically untreated cortex, the reduction of activation strength becomes gradually smaller (less negative) with lesion strength. **(C)** Left: Means ± SEM of S1 onset latencies (OL). Non-lesioned animals have longer latencies at non-BF than layer VI lesioned animals before, but not after cortical silencing. Right: Negative correlation between latency differences (OL_*non*__–__*BF*_ – OL_*BF*_) and lesion strength in layer VI (pre-muscimol). **(D)** Left: Means ± SEM of early infragranular sink (iS1) integrals. Layer VI lesioned animals have weaker sinks at BF than animals without lesions in layer VI. Right: Significant linear correlation between lesion and activation strength before muscimol treatment. **(E)** Linear correlation between relative S2 strength and lesion in layer VI. S2 is relatively stronger in layer VI lesioned animals. **(F)** No significant correlation between relative S3 strength and lesion in layer VI. Asterisk in **(C,D)** display significant group differences according to linear mixed effects model with grouped data (Supplementary Table 2A). *p < 0.05, **p < 0.01.

In both animals with and without CT lesion, acoustic stimulation with pure tones evoked a canonical cross-laminar activation pattern as already seen across species in prior studies (Metherate et al., 2005; Szymanski et al., 2009; Bollimunta et al., 2011; Schaefer et al., 2015; Brunk et al., 2019; Figure 3A, top; Figure 4A and Supplementary Figure 4). Specifically, a prominent granular sink with short latency (referred to as S1) was followed by several sinks in supra- (S2) and infragranular (S3) layers. In agreement with earlier work (Schaefer et al., 2015; Brunk et al., 2019), an early sink in deep infragranular layers (Vb/VI) could also be observed, which we refer to as iS1. In addition to the layer-specific input, we analyzed the overall columnar response by the averaged rectified CSD (AVREC) and generally observed comparable waveforms across all animal groups (Figure 3B). The time course of the AVREC waveform is characterized by a prominent early peak, which corresponds to the early thalamocortically driven input response, while later components of the AVREC curve in the non-silenced cortex (Figure 3B, black curves) resemble subsequent intracortical synaptic activity (cf. Deane et al., 2020). Accordingly, pharmacological blocking of intracortical activity by topical application of muscimol largely abolished the later sinks and sources (Figure 3A, bottom) as well as later components of the AVREC curve (Figure 3B, gray curves). As expected due to blocking of recurrent excitatory activity, muscimol led also to a significant reduction of AVREC and S1 integrals (mean reduction of 80.3% relative to the pre-value; see also Supplementary Table 3, Dep.Vars: AcS AVR BF INT, AcS S1 BF INT). This effect was independent of lesion strength (*r* = −0.184, *p* = 0.496 for lesion in layer VI; *r* = −0.399, *p* = 0.126 for layer V).

Although group-averaged CSD profiles showed generally a similar cross-laminar activation pattern, we found a trend toward a reduction of the BF-evoked prominent granular sink S1 integral with layer VI CT lesions before cortical silencing (Figure 4B, left). At non-BF as well as after cortical silencing, we did not find any relationship between lesion and activation strength. Relating non-BF activations to the associated integrals at BF, however, revealed a larger difference (percentage change) between non-BF and BF in non-lesioned compared to layer VI lesioned animals in the pharmacologically untreated cortex (Figure 4B, right panel; Supplementary Tables 1, 2A, Dep.Var: AcS_pre S1 rel_nBF INT), which suggests an impact on the TC gain of BF-evoked responses.

We also assessed the onset latency (OL) of the granular input in layer IV and found shorter OL for non-BF stimulation in layer VI lesioned animals, but no changes of the BF-evoked OL (Figure 4C left panel, Supplementary Tables 1, 2A, Dep.Vars: AcS_pre S1 nBF OL, AcS_pre S1 BF OL). When comparing OL differences of BF- and non-BF-evoked responses, we found significant differences before, but not after cortical silencing (Figure 4C, right panel; pre: Pearson’s *r* = −0.658, *p* = 0.004, post: *r* = 0.206, *p* = 0.520; Supplementary Tables 1, 2A, Dep.Var: AcS_pre S1 rel_nBF OL).

In addition, specifically the loss of layer VI CT neurons led to a significantly weaker short-latency infragranular sink iS1 at BF, but not at non-BF (Figure 4D, left panel; Supplementary Table 2A, Dep.Vars: AcS_pre iS1 BF INT, AcS_pre iS1 nBF INT). A linear correlation of the iS1 integral and the relative cell loss in layer VI was significant before cortical silencing (Figure 4D, right panel, Pearson’s *r* = 0.59, *p* = 0.013; see also Supplementary Table 1, Dep.Var: AcS_pre iS1 BF INT), and a trend in the same direction was observed after application of muscimol. The extent of layer V cell loss did not correlate with changes in the iS1 integral.

To differentiate the aforementioned effects on initial thalamocortically relayed activity from subsequent intracortical synaptic circuit processing, we analyzed the relative strengths (relative change compared to granular sink S1) of the extragranular sinks S2 and S3. At BF, the relative supragranular sink activity was stronger in layer VI lesioned animals (Supplementary Table 2A, Dep.Var: AcS_pre S2 rel_BF INT) than in cases without layer VI lesion. S2 activation increased linearly with cell loss in layer VI (Figure 4E, Person’s *r* = 0.671, *p* = 0.004). The strength of the late infragranular sink S3 did not depend on lesion efficacy (Figure 4F and Supplementary Tables 1, 2A, Dep.Var: AcS_pre S3 rel_BF INT).

### Electrically Evoked Columnar Processing

**FIGURE 5:**
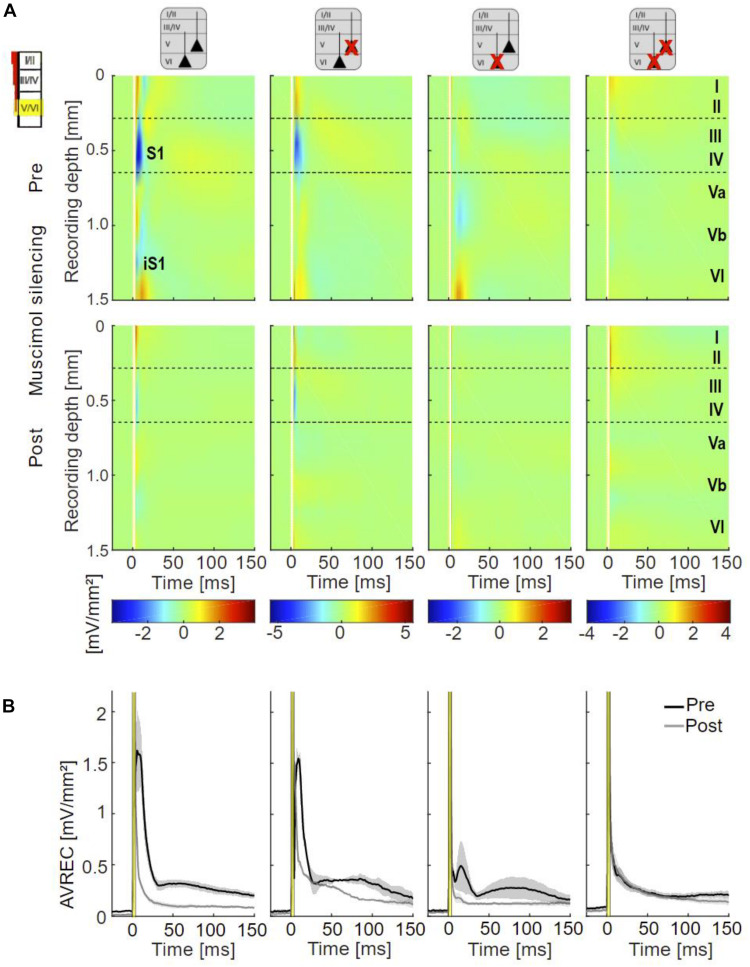
Cortical responses evoked by infragranular ICMS. **(A)** Averaged CSD profiles of all four groups (non-Les, LesV, LesVI, LesV+VI) evoked by infragranular ICMS (160 μA, stimulus artifact masked by white line), before (top) and after (bottom) application of muscimol. In the untreated condition, non (or weakly) lesioned animals show a cross-laminar activation pattern similar to that evoked by acoustic stimulation. In animals with considerable loss of layer VI neurons, S1 is much smaller than in non-lesioned animals and other rather weak sinks are observed in extragranular layers. After cortical silencing with muscimol, animals without lesions in layer VI display the two initial sinks (S1, iS1) also seen upon acoustic stimulation in the silenced cortex. In contrast, both groups with layer VI lesions lack or have only a very small S1. In the LesVI group, only weak activation remained present, while in animals with exclusive (LesV) or additional (LesV+VI) loss of layer V neurons, a stronger and longer lasting pattern of sinks and sources was observed. n_*non*__–__Les_ = 6, n_Les5_ = 2; n_LesVI_ = 4; n_LesV__+__*VI*_ = 6 (pre), 5 (post). **(B)** Grand-averaged AVREC curves evoked by layer-specific infragranular ICMS in the untreated (black) and silenced (gray) condition, showing means (solid lines) and SEM (shaded areas) of the different lesion groups. The first short peak represents the stimulus artifact (labeled by a yellow box). Before cortical silencing, strongest activation is seen in non-lesioned animals. After application of muscimol a more sustained activation was seen in animals that involved lesions in layer V, while curves of animals having no or pure layer VI lesions declined rapidly to very low values.

In non-lesioned animals, infragranular ICMS (IGstim) led to the strongest activation pattern compared to other stimulation depths (Supplementary Figure 5A). IGstim evoked a cross-laminar activation pattern similar to that seen after acoustic stimulation, i.e., a strong granular sink (S1), followed by sinks in supragranular (S2) and infragranular (S3) layers, as well as the early deep infragranular sink (iS1) (Figure 5A, top). After lesion in layer VI, electrically evoked cortical activation was generally weaker (Supplementary Figure 5). Conspicuously, S1 appeared strongly reduced in animals that included neuronal cell loss in layer VI (Figure 5A, top).

Following application of muscimol (Figure 5A, bottom), IGstim still evoked prominent sinks in the main lemniscal thalamic input layers in non-lesioned animals. In animals with lesions in layer VI (groups LesVI, LesV+VI), S1 was absent or very weak, but supra- and infragranular sinks were still present. Animals with lesions in layer V (LesV, LesV+VI) showed a comparable but slightly prolonged spatiotemporal CSD pattern compared to non-lesioned animals.

**FIGURE 6:**
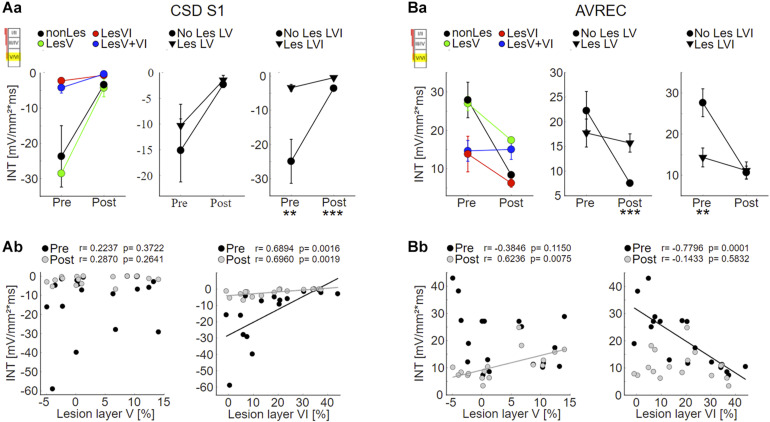
Effects of layer VI and layer V CT lesions on electrically evoked cortical activity (infragranular ICMS). **(A)** Effects on sink S1. **(Aa)** S1 means+SEM of all four groups (left), of layer V lesioned (groups LesV and LesV+VI) vs. non-layer V lesioned animals (middle), and of layer VI (groups LesVI and LesV+VI) vs. non-layer VI lesioned animals (right). **(Ab)** Individual S1 INT plotted against lesion strengths in layer V (left) or VI (right), before (black) and after (gray) application of muscimol. Animals with loss of layer VI CT neurons had weaker (less negative) granular sinks, both before and after cortical silencing. **(B)** Effects on AVRECs. **(Ba)** AVREC group means and **(Bb)** scatterplots as described for (A). Before silencing, strongest activation was evoked in animals without cell loss in layer VI. After application of muscimol, highest activity was seen in animals having lesions in layer V. Significant group comparisons **(Aa,Ba)** using a linear mixed effects model (Supplementary Table 2A) are labeled by asterisks (*p < 0.05, **p < 0.01, ***p < 0.001).

As expected based on the qualitative assessment, statistical analysis revealed that the strength of S1 evoked by infragranular ICMS decreased with increasing photolysis in layer VI, but not layer V, both before and after application of muscimol (Figure 6Ab; LesVI pre: Pearson’s *r* = 0.689, *p* = 0.0015; LesVI post: *r* = 0.696, *p* = 0.0019, see also Supplementary Tables 1, 2A, Dep.Var: ICMS S1 IG INT). Thereby, S1 was generally smaller after cortical silencing (Figure 6A and Supplementary Table 3, Dep.Var: ICMS S1 IG INT).

Also the averaged AVREC curves suggest that before cortical silencing, the strongest activation is produced in non/weakly lesioned animals (Figure 5B, black curves). This is statistically supported by a significant correlation between integrals of the AVREC and lesion strength of layer VI (Figure 6Bb, right panel, Pearson’s *r* = −0.78, *p* = 1.37^∗^10^–4^; Supplementary Table 1, Dep.Var: ICMS AVR IG INT) and a significantly different mean of AVREC integrals (Figure 6Ba and Table 3).

After cortical silencing (Figure 5B, gray curves), IGstim produced a short activation in non/weakly lesioned animals. Curves of animals that included lesions in layer V, however, in accordance with the before described CSD pattern, showed a more sustained, slowly decaying activation that had similar amplitudes than the curves of these animals before silencing. Mean plots (Figure 6Ba and Supplementary Table 2A, Dep.Var: ICMS AVR IG INT) and correlation analyses (Figure 6Bb, left panel, Pearson’s *r* = 0.624, *p* = 0.0075; Supplementary Figure 5B and Supplementary Table 1, Dep.Var: ICMS AVR IG INT) between lesion strength in layer V and the INTs of AVREC underpin the observation, that after cortical silencing animals with layer V lesions displayed higher activation than those without (Supplementary Table 3, Dep.Var: ICMS AVR IG INT).

## Discussion

In this study, we have focused on the corticofugal output system, originating in the infragranular cortical layers and influencing subcortical targets thereby affecting sensory, motor and cognitive functions (Williamson and Polley, 2019; Clayton et al., 2020; Prasad et al., 2020). We lesioned CT projection neurons in the primary ACx of anesthetized Mongolian gerbils, while leaving all other components of the TC and intracortical feedforward circuitry intact. Whereas lesion of CT neurons in layer V had no or only moderate effects on tone or ICMS-evoked cortical processing, lesion of CT neurons in layer VI led to layer-specific changes of the tone-evoked spatiotemporal cortical activity profile with a reduced input gain for preferred frequency input. By direct ICMS of corticoefferent output circuits in deeper layers, we furthermore revealed circuit-specific effects of lesioning layer VI CT neurons. Electrically evoked columnar responses in the intact ACx mimicked the spatiotemporal cascade of synaptic activity during auditory processing. Lesion of layer VI neurons led to a profound reduction of electrically evoked overall translaminar activity, confirming the hypothesis of recurrent extracortical feedback originating from the corticothalamic circuitry after cortical stimulation (Happel et al., 2014). Our study thereby suggests an important role of particularly the CT feedback neurons in layer VI in orchestrating the feedforward translaminar information flow in auditory cortex via recurrent CT feedback.

### Lesion Specificity of Corticothalamic Neurons

Lesion specificity was determined based on the location of retrograde fluorescent labeling of neuronal somata or by laser-induced cell loss (NeuN-stained slices) in relation to the thalamic injection site. While labeling was particularly high in layers VI after injections into MGv, labeling of layer V neurons in auditory cortical fields AI and AAF was stronger after terminals in MGd or MGm had incorporated the beads. We targeted mainly the lemniscal thalamus with our injections, where layer VI CT neurons are likely to constitute a larger portion of the total target cells compared to layer V CT neurons (Williamson and Polley, 2019). In accordance, cell loss in layer VI was up to 40% and in layer V max. 15%. Based on the available literature, the observed reduced cell density in layer VI corresponds to a reduction of approx. 80% of CT neurons of layer VI (Kelly and Wong, 1981; Prieto and Winer, 1999; Homma et al., 2017). The distinct lesion patterns are in accordance with previous reports linking layer VI CT neurons more to the lemniscal auditory pathway, while layer V corticofugal neurons project less specifically to non-lemniscal and other subcortical target areas (Alitto and Usrey, 2003; Briggs and Usrey, 2007; Usrey and Sherman, 2019; Williamson and Polley, 2019). It has been suggested that layer V corticofugal neurons broadcast sensory information to distributed non-lemniscal targets, while layer VI CT neurons with their more lemniscal interconnectivity are ideal regulators of the local TC gain control (Llano and Sherman, 2009; Olsen et al., 2012; Guo et al., 2017; Williamson and Polley, 2019).

### Corticothalamic Gain Control of Layer-Specific Subcortical Input

Deeper layer CT neurons are glutamate-releasing pyramidal neurons (Bortone et al., 2014) and have strong local intracortical connections spanning all cortical layers (Guo et al., 2017; Baker et al., 2018). In order to observe their impact on the overall columnar response characteristics, we used laminar multichannel CSD recordings across all cortical laminae giving rise to layer-specific synaptic population activity. We have used physiological stimulation of the primary ACx via its primarily lemniscal input system, and direct artificial stimulation of the cortical network by layer-specific ICMS bypassing the bottom-up pathway. Selective elimination of CT neurons in layer VI, but not layer V led to reduced initial tone-evoked synaptic activity in cortical layer Vb/VI quantified by a reduced current flow of the thalamocortically relayed sink iS1 (Szymanski et al., 2009; Brunk et al., 2019; Deane et al., 2020). Also, we observed less specific frequency processing in terms of the relative input strength and onset latency of sink S1. We further found increased subsequent intracortical activity in supragranular layers (sink S2) correlating with the lesion efficacy in layer VI. In contrast, synaptic current flow in upper layer Va (sink S3) was not affected by lesioning cells in layer VI. Lesion of layer V CT neurons did not affect the columnar current flow in a significant manner. This data suggests that layer VI CT neurons play a pivotal role for a local gain control of TC inputs and their integration with broader spectral inputs relayed via upper layers at a given cortical patch (Larkum, 2013; Brunk et al., 2019; Zempeltzi et al., 2020). We observed these effects after a physical lesion of CT layer VI neurons, which, as circuit manipulations in general, may lead to short-term and compensatory circuit adaptations (Otchy et al., 2015). However, recent work has revealed a particular function of layer VI CT neurons in frequency integration in agreement with our findings (Guo et al., 2017). We further used direct ICMS in order to perform a detailed circuit analysis of potential contributions of recurrent cortico-subcortical loops to the observed network effects. In non-lesioned animals, infragranular ICMS evoked feedforward synaptic activity patterns with translaminar information flow in agreement with previous reports (Lison et al., 2013; Happel et al., 2014). Effective lesioning of layer VI neurons strongly reduced the translaminar activation cascade, which was not observed when lesioning layer V cells. In a previous study, we have hypothesized that recurrent CT feedback recruited by infragranular layer stimulation routes back to granular input layers of the ACx (Happel et al., 2014) most likely via the MGv (Saldeitis et al., 2014). This granular feedback signal thereby may trigger granular synaptic recurrent excitation regulating TC gain control and hence initiate translaminar activity patterns (Liu et al., 2007; Deane et al., 2020). In agreement, lesion strength of layer VI neurons was negatively correlated with synaptic input in granular layers III/IV quantified by the integral of the dominant current sink S1 before cortical silencing, and further with an impaired translaminar activation cascade. The residual granular activation after cortical silencing was likewise diminished in animals with a layer VI lesion (cf. Happel et al., 2014). Henceforth, our data corroborate the hypothesis that layer VI CT neurons are the cellular substrate for this recurrent and excitatory loop and are an essential circuit element for ICMS-generated columnar activity patterns. Whether our finding is alternatively due to the eliminated intracortical collaterals of these layer VI neurons (Oviedo et al., 2010; Kratz and Manis, 2015), or a mixture of diminished intracortical and cortico-thalamo-cortical activation, cannot be resolved with our experimental design and would need further investigation. In a similar way, the less prominent effects on ICMS-evoked responses after lesioning layer V neurons may also arise via different possible changes of the circuit processing.

Our study investigated effects of CT lesion in anesthetized gerbils. It therefore remains open, how CT feedback from layers V and VI would affect auditory perception and behavior. It was shown before, that particularly layer VI CT neurons are involved in auditory detection and discrimination behavior (Guo et al., 2017).

### Perspectives in Corticofugal Pathway Research

Corticothalamic feedback originating from layer VI neurons acts more locally, while the system originating in layer V acts more globally. Layer V neurons project to various downstream target regions including other cortices or subcortical regions, such as the striatum. They have been linked to sensorimotor integration, sensory-guided movement control (Znamenskiy and Zador, 2013; Guo et al., 2017), attentive functions (Yu et al., 2004) and might also receive inhibition from corollary discharges (Schneider et al., 2014). Lesioning of these neurons may have appeared as the prolonged electrically evoked activity after cortical silencing, because their apoptosis probably affected both lemniscally (LesV only) and non-lemniscally (LesV+VI) driven TC input (see Figure 5). We hypothesize that a release from inhibition of otherwise sustained firing neurons in subcortical nuclei such as the inferior colliculus (Smith, 1992; Sivaramakrishnan and Oliver, 2001), which may normally be mediated by layer V CT neurons sending axon collaterals to the IC (Asokan et al., 2018), plays a role for the prolonged cortical activation. Before cortical silencing, this activity may remain hidden due to intracortical inhibitory mechanisms. However, more animals with CT lesions restricted to layer V are required to ultimately confirm and interpret this effect.

Dopaminergic synapses have been preferentially found in infragranular layers of ACx (Schicknick et al., 2008). Further studies need to reveal how dopaminergic neuromodulation may affect the different corticofugal cell types, as it has been shown that deeper layers specifically integrate sensory and reward-related signals in the sensory cortex in the potential service of action-outcome integration and adaptive coding of expectancy (Mylius et al., 2014; Happel, 2016; Brunk et al., 2019).

## Data Availability Statement

The raw data supporting the conclusions of this article will be made available by the authors, without undue reservation.

## Ethics Statement

All experiments were conducted in accordance with the international NIH Guidelines for Animals in Research and with ethical standards for the care and use of animals in research defined by the German Law for the protection of experimental animals. Experiments were approved by an ethics committee of the state Saxony-Anhalt, Germany.

## Author Contributions

KS carried out the experiments and data analysis. MH and MJ supervised the experiments and data analysis. MH and KS wrote the first draft of the manuscript. All authors finalized the manuscript and designed the experiments.

## Conflict of Interest

The authors declare that the research was conducted in the absence of any commercial or financial relationships that could be construed as a potential conflict of interest.

## Footnotes

1 http://imagej.nih.gov/ij/
