# Supplementary material for: Laser-Induced Apoptosis of Corticothalamic Neurons in Layer VI of Auditory Cortex Impact on Cortical Frequency Processing
Source: Front Neural Circuits. 2021 Jul 12;15:659280. doi: 10.3389/fncir.2021.659280 (PMC8311662; doi:10.3389/fncir.2021.659280)
Supplement: Supplementary file 1 [file Data_Sheet_1.docx]

**Supplementary Material**

**Supplementary Figures**

***
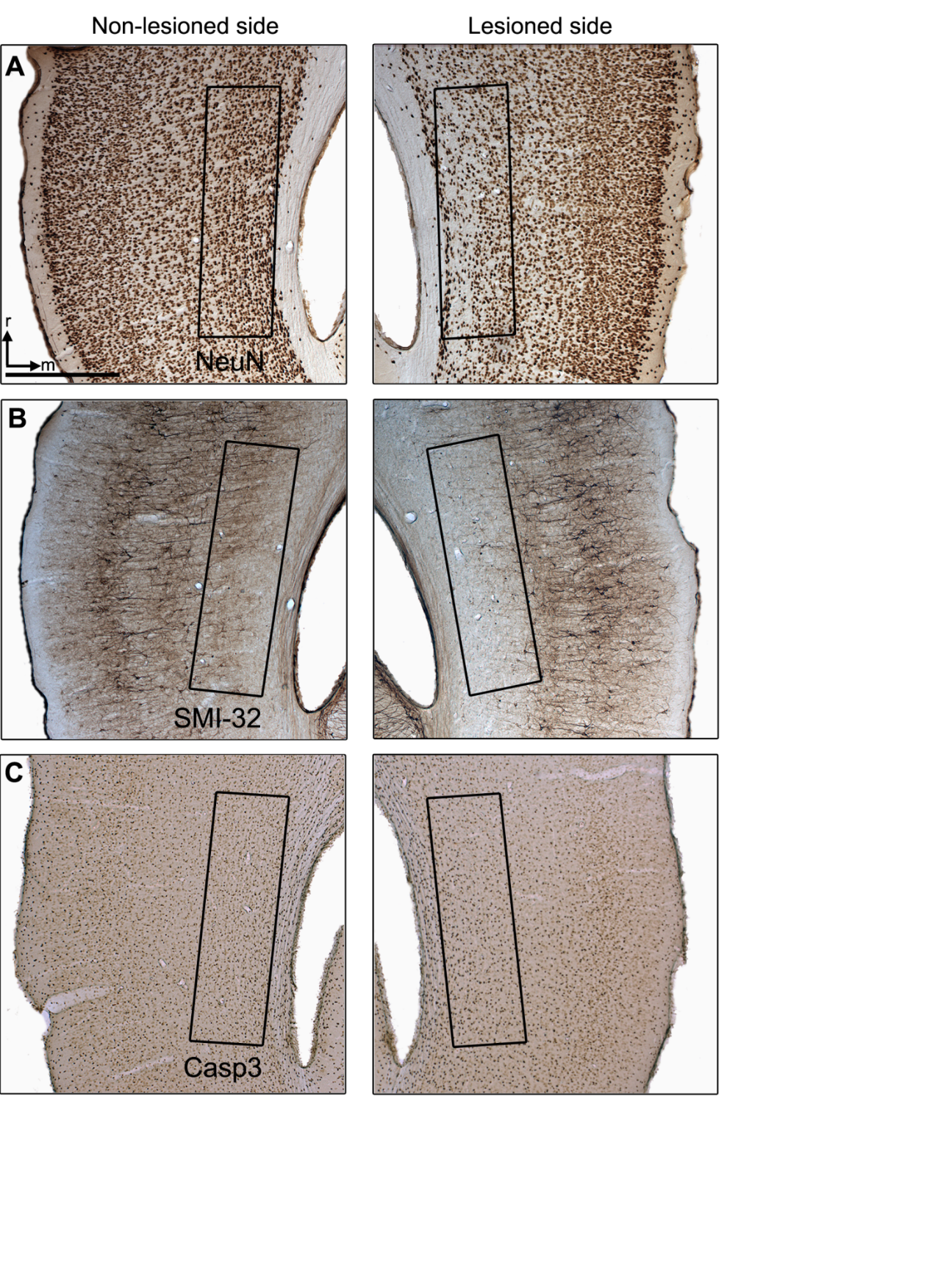
***

**Supplementary Figure 1.** Three immunohistochemical stains to display neuronal cell loss or apoptotic cells, evaluated by comparing the non-lesioned (left) with the lesioned (right) cortical sides of AI. **(A)** Based on its clear visualization of neuronal nuclei, we regarded the NeuN stain well-suited for quantification of neuronal cell loss. **(B)** An antibody that recognizes a nonphosphorylated epitope of neurofilament H (SMI-32) preferentially visualizes pyramidal neurons in a Golgi-like manner. This marker revealed neuronal loss in deep infragranular layers of the lesioned side, particularly recognizable by the reduction of neuronal processes, but quantification was therefore difficult. **(C)** Caspase 3 is a key enzyme involved in the apoptotic cascade. Active Caspase 3 (Casp3) assay did not provide differences between both cortical sides, indicating that photolytically induced apoptosis was already completed by the time of euthanasia. Scale bar: 500 μm.


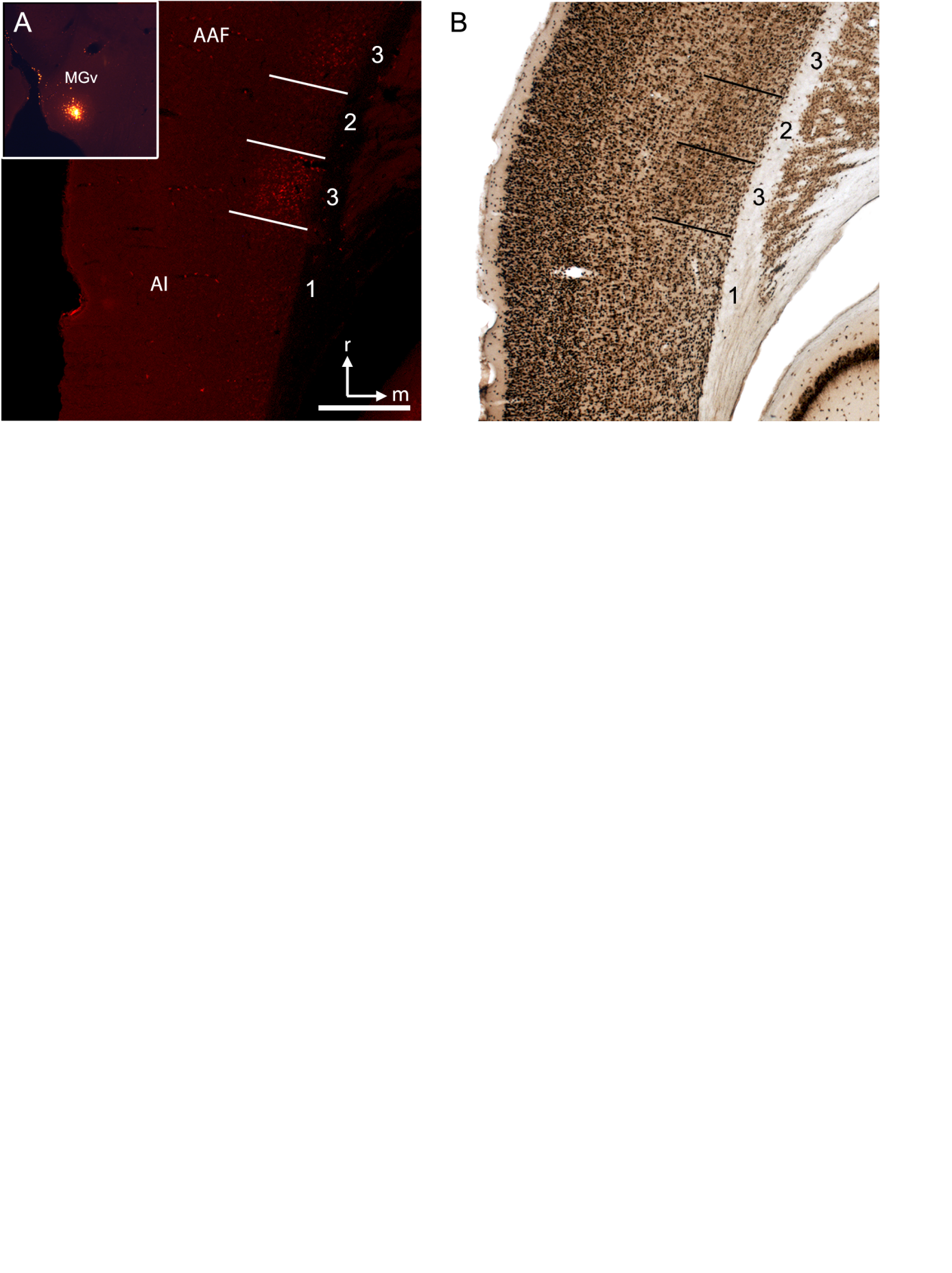


**Supplementary Figure 2.** The specificity of the photolytic apoptosis (i.e. successful transport of red retrobeads to infragranular layers of ACx, successful attachment of photolytic component, efficacy and specificity of laser illumination; see also main Figure 1C) was revealed by complementary analyses of fluorescent **(A)** and NeuN-stained slices **(B)** of the ACx. Regions that were labeled, such as layer VI of the AAF (A, zone 3), but not illuminated, did not show neuronal degeneration (B, zone 3). Cells within regions that were not labeled (rostral AI, zone 2) were also preserved. Neuronal loss, i.e. zones with reduced cell density in layer VI (B, zone 1), was generally accompanied by bleaching in the correspondent fluorescent slices (A, zone 1). Scale bar: 500 μm.


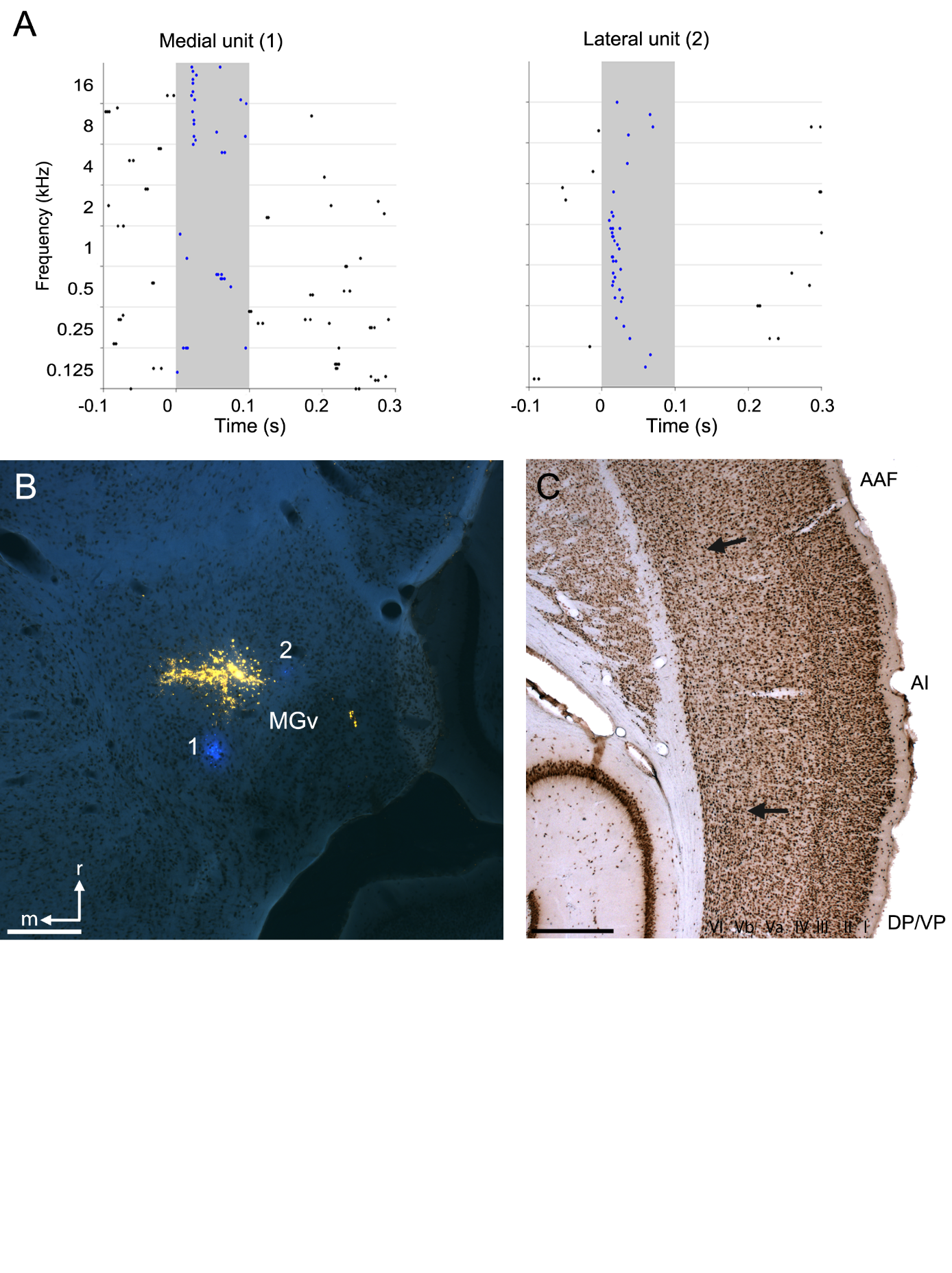


**Supplementary Figure 3.** Responsiveness of thalamic neurons near the injection site after photolytic apoptosis of CT neurons. **(A)** To ensure that the chlorin e6-conjugated retrobeads and the laser exposure do not interfere with the activity of nearby thalamic neurons, acoustically evoked responses were recorded with a tungsten electrode vertically inserted into MGv of an anesthetized animal. The rasterplots show tonotopic responses of MGv neurons adjacent to the injections site (B) (medial unit (1): 8-16 kHz, lateral unit (2) 0.5-1 kHz; pure tones from 125 Hz to 16 kHz, 54 dB SPL, stimulus duration 100 ms, 10 repetitions). **(B)** NeuN-stained horizontal slice showing the recording sites 1 and 2 (labeled by Fast Blue), and the injection site (chlorin e6 conjugated retrobeads, appearing yellowish due to the applied filter set (F31-013, AHF Analysentechnik AG, Germany). **(C)** NeuN-stained slice representing reduced cell density in layer VI of mid frequency regions of the ipsilateral AI (area enclosed by arrows) of the same animal, consistent with the intrathalamic location of the injection site. Scale bars: 300 μm (B), 500 μm (C).


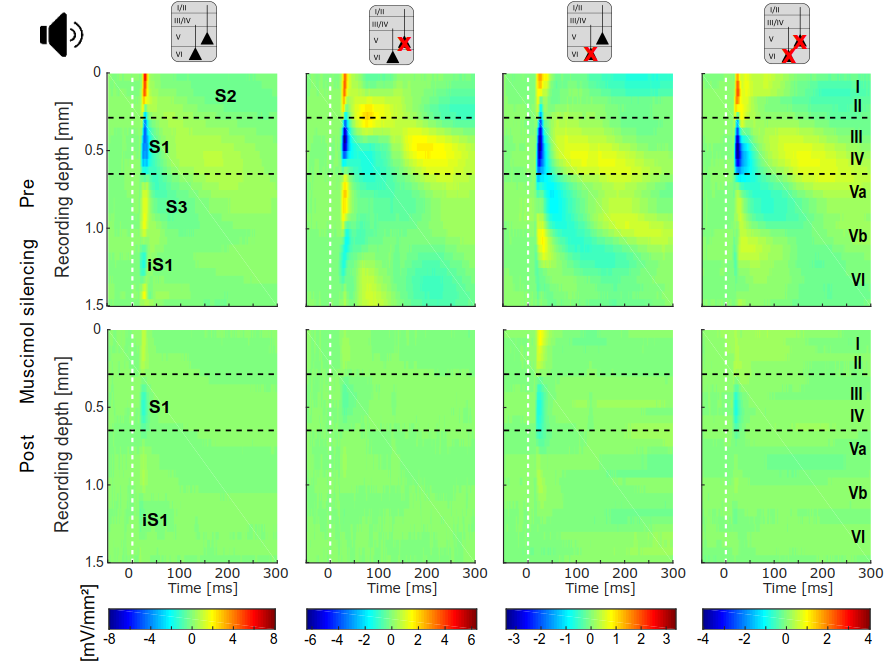


**Supplementary Figure 4.** Averaged CSD profiles of all four groups (nonLes, LesV, LesVI, LesV+VI) evoked by acoustic stimulation at BF (54 dB SPL), before (top) and after (bottom) application of muscimol. In the untreated condition, lesioned animals show a cross-laminar activation pattern similar to that of non-lesioned animals. However, in animals with considerable loss of layer VI neurons, iS1 is smaller than in non-lesioned animals. After cortical silencing with muscimol, activation is weaker in all groups and only the initial sinks S1 and iS1 remain present. n_nonLes_ = 6, n_LesV_ = 1, n_LesVI_ = 4; n_LesV+VI_ = 6 (pre), 5 (post).


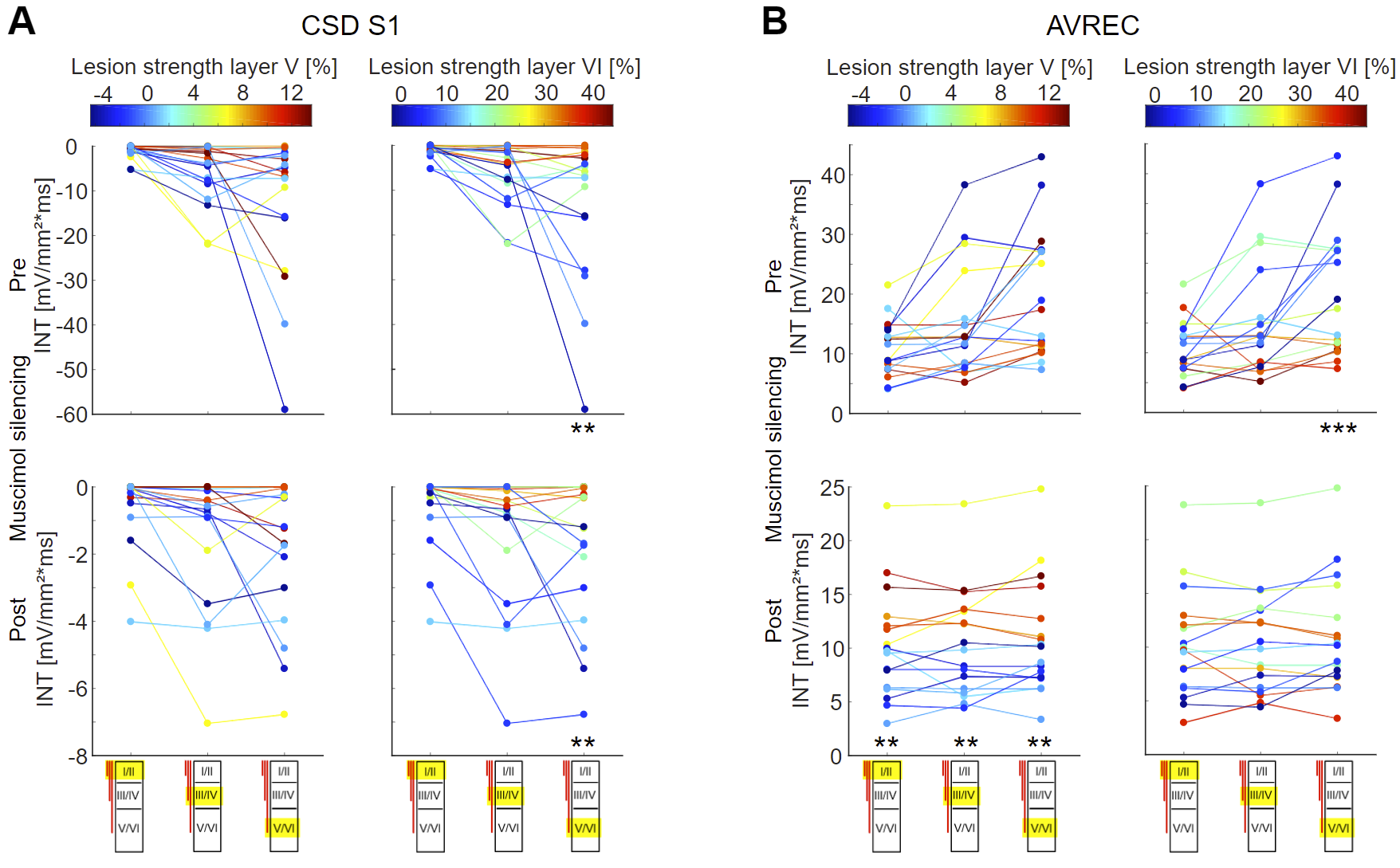


**Supplementary Figure 5. Effects of layer V and layer VI CT lesions and stimulus depth of ICMS on cortical activation. (A)** Diagrams depicting the integrals of S1 evoked by layer-specific ICMS (160 μA), before and after cortical silencing. Data points are color-coded according to their respective lesion strength in layers V (left) or VI (right). Animals with loss of layer VI CT neurons had weaker (less negative) granular sinks, particularly upon IGstim, both before and after cortical silencing. The strength of S1 did not depend on lesions in layer V. **(B)** Diagrams depicting the individual integrals of AVRECs. Before silencing, strongest overall activation was evoked by IGstim in animals without cell loss in layer VI. After application of muscimol, highest activation was seen in animals having lesions in layer V (ordered color-coded values, independent of stimulus depth). Significant linear corrleations between activation and lesion strength are labeled by asterisks (*p<0.05, **p<0.01, ***p<0.001).

**Supplementary Tables**

**Suppl. Table 1: Linear mixed-effects model analysis of layer specific lesion effects using individual percentage cell loss; pre and post muscimol separately**

| **Predictor** | **Estimate** | **SE** | **tStat** | **DF** | **pValue** | **Lower** | **Upper** | **Dependent Variable** |
| --- | --- | --- | --- | --- | --- | --- | --- | --- |
| (Intercept) | 38,33 | 6,97 | 5,50 | 14 | 0,0001 | 23,39 | 53,27 | AcS_pre AVR BF INT |
| LesV | -0,06 | 0,68 | -0,09 | 14 | 0,9290 | -1,52 | 1,40 | AcS_pre AVR BF INT |
| LesVI | -0,34 | 0,32 | -1,07 | 14 | 0,3018 | -1,02 | 0,34 | AcS_pre AVR BF INT |
| (Intercept) | 16,34 | 0,53 | 31,08 | 12 | 7,74E-13 | 15,19 | 17,48 | AcS_pre AVR BF OL |
| LesV | 0,04 | 0,05 | 0,75 | 12 | 0,4675 | -0,08 | 0,16 | AcS_pre AVR BF OL |
| LesVI | 0,03 | 0,02 | 1,25 | 12 | 0,2335 | -0,02 | 0,08 | AcS_pre AVR BF OL |
| (Intercept) | -78,04 | 13,58 | -5,75 | 14 | 0,0001 | -107,15 | -48,92 | AcS_pre S1 BF INT |
| LesV | -1,24 | 1,33 | -0,93 | 14 | 0,3669 | -4,08 | 1,61 | AcS_pre S1 BF INT |
| LesVI | 1,17 | 0,62 | 1,89 | 14 | 0,0803 | -0,16 | 2,49 | AcS_pre S1 BF INT |
| (Intercept) | 19,58 | 0,60 | 32,55 | 14 | 1,35E-14 | 18,29 | 20,87 | AcS_pre S1 BF OL |
| LesV | 0,21 | 0,06 | 3,55 | 14 | **0,0032** | 0,08 | 0,33 | AcS_pre S1 BF OL |
| LesVI | -0,04 | 0,03 | -1,58 | 14 | 0,1374 | -0,10 | 0,02 | AcS_pre S1 BF OL |
| (Intercept) | -13,54 | 4,45 | -3,04 | 14 | 0,0088 | -23,08 | -4,00 | AcS_pre S1 nBF INT |
| LesV | -0,17 | 0,43 | -0,40 | 14 | 0,6981 | -1,10 | 0,76 | AcS_pre S1 nBF INT |
| LesVI | 0,03 | 0,20 | 0,14 | 14 | 0,8932 | -0,41 | 0,46 | AcS_pre S1 nBF INT |
| (Intercept) | 27,90 | 0,98 | 28,55 | 14 | 8,24E-14 | 25,81 | 30,00 | AcS_pre S1 nBF OL |
| LesV | 0,26 | 0,10 | 2,73 | 14 | **0,0162** | 0,06 | 0,47 | AcS_pre S1 nBF OL |
| LesVI | -0,19 | 0,04 | -4,17 | 14 | **0,0009** | -0,28 | -0,09 | AcS_pre S1 nBF OL |
| (Intercept) | -87,47 | 3,68 | -23,80 | 14 | 1,01E-12 | -95,35 | -79,58 | AcS_pre S1 rel_nBF INT |
| LesV | -0,40 | 0,36 | -1,12 | 14 | 0,2829 | -1,17 | 0,37 | AcS_pre S1 rel_nBF INT |
| LesVI | 0,66 | 0,17 | 3,93 | 14 | **0,0015** | 0,30 | 1,02 | AcS_pre S1 rel_nBF INT |
| (Intercept) | 8,32 | 0,88 | 9,42 | 14 | 1,95E-07 | 6,43 | 10,22 | AcS_pre S1 rel_nBF OL |
| LesV | 0,05 | 0,09 | 0,60 | 14 | 0,5563 | -0,13 | 0,24 | AcS_pre S1 rel_nBF OL |
| LesVI | -0,14 | 0,04 | -3,54 | 14 | **0,0033** | -0,23 | -0,06 | AcS_pre S1 rel_nBF OL |
| (Intercept) | -21,64 | 3,88 | -5,58 | 14 | 0,0001 | -29,95 | -13,33 | AcS_pre iS1 BF INT |
| LesV | 0,14 | 0,38 | 0,36 | 14 | 0,7247 | -0,68 | 0,95 | AcS_pre iS1 BF INT |
| LesVI | 0,45 | 0,18 | 2,56 | 14 | **0,0226** | 0,07 | 0,83 | AcS_pre iS1 BF INT |
| (Intercept) | 16,14 | 1,73 | 9,31 | 12 | 7,74E-07 | 12,36 | 19,92 | AcS_pre iS1 BF OL |
| LesV | -0,07 | 0,19 | -0,39 | 12 | 0,7022 | -0,48 | 0,33 | AcS_pre iS1 BF OL |
| LesVI | 0,13 | 0,08 | 1,65 | 12 | 0,1251 | -0,04 | 0,30 | AcS_pre iS1 BF OL |
| (Intercept) | -5,98 | 1,94 | -3,08 | 14 | 0,0081 | -10,14 | -1,82 | AcS_pre iS1 nBF INT |
| LesV | 0,08 | 0,19 | 0,42 | 14 | 0,6844 | -0,33 | 0,49 | AcS_pre iS1 nBF INT |
| LesVI | 0,02 | 0,09 | 0,22 | 14 | 0,8282 | -0,17 | 0,21 | AcS_pre iS1 nBF INT |
| (Intercept) | 21,83 | 2,73 | 7,99 | 13 | 2,26E-06 | 15,93 | 27,73 | AcS_pre iS1 nBF OL |
| LesV | -0,26 | 0,28 | -0,93 | 13 | 0,3674 | -0,88 | 0,35 | AcS_pre iS1 nBF OL |
| LesVI | 0,08 | 0,12 | 0,64 | 13 | 0,5344 | -0,19 | 0,35 | AcS_pre iS1 nBF OL |
| (Intercept) | -57,47 | 29,60 | -1,94 | 13 | 0,0741 | -121,41 | 6,47 | AcS_pre S2 BF INT |
| LesV | -0,35 | 2,93 | -0,12 | 13 | 0,9055 | -6,68 | 5,97 | AcS_pre S2 BF INT |
| LesVI | -1,66 | 1,36 | -1,22 | 13 | 0,2445 | -4,61 | 1,28 | AcS_pre S2 BF INT |
| (Intercept) | 19,09 | 2,02 | 9,45 | 11 | 1,30E-06 | 14,64 | 23,54 | AcS_pre S2 BF OL |
| LesV | 0,00 | 0,23 | -0,02 | 11 | 0,9865 | -0,52 | 0,51 | AcS_pre S2 BF OL |
| LesVI | 0,04 | 0,10 | 0,46 | 11 | 0,6545 | -0,17 | 0,26 | AcS_pre S2 BF OL |
| (Intercept) | -50,12 | 47,69 | -1,05 | 13 | 0,3124 | -153,14 | 52,91 | AcS_pre S2 rel_BF INT |
| LesV | 0,12 | 4,72 | 0,03 | 13 | 0,9793 | -10,07 | 10,32 | AcS_pre S2 rel_BF INT |
| LesVI | 7,29 | 2,20 | 3,32 | 13 | **0,0056** | 2,54 | 12,05 | AcS_pre S2 rel_BF INT |
| (Intercept) | -55,28 | 13,59 | -4,07 | 14 | 0,0012 | -84,42 | -26,14 | AcS_pre S3 BF INT |
| LesV | -0,59 | 1,33 | -0,45 | 14 | 0,6623 | -3,44 | 2,25 | AcS_pre S3 BF INT |
| LesVI | -0,18 | 0,62 | -0,30 | 14 | 0,7712 | -1,51 | 1,15 | AcS_pre S3 BF INT |
| (Intercept) | 19,09 | 2,02 | 9,45 | 11 | 1,30E-06 | 14,64 | 23,54 | AcS_pre S3 BF OL |
| LesV | 0,00 | 0,23 | -0,02 | 11 | 0,9865 | -0,52 | 0,51 | AcS_pre S3 BF OL |
| LesVI | 0,04 | 0,10 | 0,46 | 11 | 0,6545 | -0,17 | 0,26 | AcS_pre S3 BF OL |
| (Intercept) | -15,56 | 32,59 | -0,48 | 14 | 0,6404 | -85,45 | 54,33 | AcS_pre S3 rel_BF INT |
| LesV | 0,98 | 3,18 | 0,31 | 14 | 0,7617 | -5,84 | 7,81 | AcS_pre S3 rel_BF INT |
| LesVI | 2,13 | 1,49 | 1,43 | 14 | 0,1739 | -1,06 | 5,32 | AcS_pre S3 rel_BF INT |
| (Intercept) | 8,35 | 2,26 | 3,69 | 13 | 0,0027 | 3,46 | 13,23 | AcS_post AVR BF INT |
| LesV | -0,12 | 0,22 | -0,57 | 13 | 0,5814 | -0,60 | 0,35 | AcS_post AVR BF INT |
| LesVI | -0,04 | 0,11 | -0,37 | 13 | 0,7150 | -0,27 | 0,19 | AcS_post AVR BF INT |
| (Intercept) | 17,01 | 1,02 | 16,61 | 13 | 3,90E-10 | 14,79 | 19,22 | AcS_post AVR BF OL |
| LesV | 0,15 | 0,10 | 1,53 | 13 | 0,1509 | -0,06 | 0,37 | AcS_post AVR BF OL |
| LesVI | -0,01 | 0,05 | -0,11 | 13 | 0,9180 | -0,11 | 0,10 | AcS_post AVR BF OL |
| (Intercept) | -17,21 | 4,81 | -3,58 | 13 | 0,0034 | -27,59 | -6,83 | AcS_post S1 BF INT |
| LesV | 0,04 | 0,47 | 0,09 | 13 | 0,9334 | -0,97 | 1,05 | AcS_post S1 BF INT |
| LesVI | 0,22 | 0,23 | 0,97 | 13 | 0,3474 | -0,27 | 0,71 | AcS_post S1 BF INT |
| (Intercept) | 18,55 | 0,79 | 23,37 | 13 | 5,28E-12 | 16,83 | 20,26 | AcS_post S1 BF OL |
| LesV | 0,17 | 0,08 | 2,15 | 13 | 0,0512 | 0,00 | 0,33 | AcS_post S1 BF OL |
| LesVI | -0,02 | 0,04 | -0,44 | 13 | 0,6699 | -0,10 | 0,06 | AcS_post S1 BF OL |
| (Intercept) | -4,38 | 2,02 | -2,17 | 12 | 0,0512 | -8,78 | 0,03 | AcS_post S1 nBF INT |
| LesV | -0,17 | 0,22 | -0,77 | 12 | 0,4562 | -0,66 | 0,32 | AcS_post S1 nBF INT |
| LesVI | 0,07 | 0,10 | 0,73 | 12 | 0,4815 | -0,14 | 0,29 | AcS_post S1 nBF INT |
| (Intercept) | 22,62 | 1,74 | 12,97 | 9 | 3,95E-07 | 18,68 | 26,57 | AcS_post S1 nBF OL |
| LesV | 0,14 | 0,22 | 0,64 | 9 | 0,5372 | -0,36 | 0,65 | AcS_post S1 nBF OL |
| LesVI | 0,07 | 0,08 | 0,86 | 9 | 0,4132 | -0,11 | 0,26 | AcS_post S1 nBF OL |
| (Intercept) | -80,45 | 6,30 | -12,77 | 12 | 2,41E-08 | -94,18 | -66,72 | AcS_post S1 rel_nBF INT |
| LesV | 0,46 | 0,70 | 0,65 | 12 | 0,5266 | -1,07 | 1,98 | AcS_post S1 rel_nBF INT |
| LesVI | 0,21 | 0,31 | 0,68 | 12 | 0,5094 | -0,46 | 0,88 | AcS_post S1 rel_nBF INT |
| (Intercept) | 5,65 | 1,51 | 3,73 | 9 | 0,0047 | 2,22 | 9,08 | AcS_post S1 rel_nBF OL |
| LesV | 0,12 | 0,19 | 0,64 | 9 | 0,5406 | -0,31 | 0,56 | AcS_post S1 rel_nBF OL |
| LesVI | 0,03 | 0,07 | 0,43 | 9 | 0,6767 | -0,13 | 0,19 | AcS_post S1 rel_nBF OL |
| (Intercept) | -77,41 | 4,34 | -17,85 | 13 | 1,59E-10 | -86,77 | -68,04 | AcS_post S1 rel_preBF INT |
| LesV | -0,66 | 0,42 | -1,57 | 13 | 0,1415 | -1,58 | 0,25 | AcS_post S1 rel_preBF INT |
| LesVI | -0,05 | 0,20 | -0,24 | 13 | 0,8103 | -0,49 | 0,39 | AcS_post S1 rel_preBF INT |
| (Intercept) | -6,73 | 1,97 | -3,42 | 13 | 0,0046 | -10,98 | -2,47 | AcS_post iS1 BF INT |
| LesV | 0,05 | 0,19 | 0,24 | 13 | 0,8159 | -0,37 | 0,46 | AcS_post iS1 BF INT |
| LesVI | 0,16 | 0,09 | 1,76 | 13 | 0,1023 | -0,04 | 0,36 | AcS_post iS1 BF INT |
| (Intercept) | 14,03 | 3,80 | 3,70 | 10 | 0,0041 | 5,58 | 22,49 | AcS_post iS1 BF OL |
| LesV | -0,06 | 0,41 | -0,16 | 10 | 0,8782 | -0,98 | 0,85 | AcS_post iS1 BF OL |
| LesVI | 0,36 | 0,18 | 1,96 | 10 | 0,0785 | -0,05 | 0,77 | AcS_post iS1 BF OL |
| (Intercept) | -1,84 | 0,74 | -2,49 | 12 | 0,0283 | -3,46 | -0,23 | AcS_post iS1 nBF INT |
| LesV | -0,04 | 0,08 | -0,51 | 12 | 0,6165 | -0,22 | 0,14 | AcS_post iS1 nBF INT |
| LesVI | 0,04 | 0,04 | 1,22 | 12 | 0,2450 | -0,03 | 0,12 | AcS_post iS1 nBF INT |
| (Intercept) | 16,61 | 2,93 | 5,68 | 4 | 0,0048 | 8,49 | 24,73 | AcS_post iS1 nBF OL |
| LesV | -2,05 | 0,60 | -3,41 | 4 | **0,0271** | -3,72 | -0,38 | AcS_post iS1 nBF OL |
| LesVI | 1,07 | 0,31 | 3,46 | 4 | **0,0259** | 0,21 | 1,92 | AcS_post iS1 nBF OL |
| (Intercept) | 10,55 | 1,81 | 5,82 | 15 | 3,39E-05 | 6,69 | 14,42 | ICMS_pre AVR SG INT |
| LesV | 0,03 | 0,18 | 0,15 | 15 | 0,8815 | -0,36 | 0,42 | ICMS_pre AVR SG INT |
| LesVI | 0,01 | 0,08 | 0,13 | 15 | 0,8989 | -0,17 | 0,19 | ICMS_pre AVR SG INT |
| (Intercept) | 19,99 | 3,24 | 6,17 | 15 | 1,78E-05 | 13,09 | 26,90 | ICMS_pre AVR G INT |
| LesV | -0,29 | 0,33 | -0,90 | 15 | 0,3821 | -0,99 | 0,40 | **ICMS_pre AVR G INT** |
| LesVI | -0,20 | 0,15 | -1,35 | 15 | 0,1978 | -0,52 | 0,12 | ICMS_pre AVR G INT |
| (Intercept) | 31,54 | 2,60 | 12,15 | 15 | 0,0000 | 26,01 | 37,07 | ICMS_pre AVR IG INT |
| LesV | -0,15 | 0,26 | -0,58 | 15 | 0,5735 | -0,71 | 0,41 | ICMS_pre AVR IG INT |
| LesVI | -0,56 | 0,12 | -4,67 | 15 | **0,0003** | -0,81 | -0,30 | ICMS_pre AVR IG INT |
| (Intercept) | -1,93 | 0,58 | -3,31 | 15 | 0,0048 | -3,18 | -0,69 | ICMS_pre S1 SG INT |
| LesV | 0,07 | 0,06 | 1,20 | 15 | 0,2483 | -0,05 | 0,20 | ICMS_pre S1 SG INT |
| LesVI | 0,03 | 0,03 | 1,16 | 15 | 0,2626 | -0,03 | 0,09 | ICMS_pre S1 SG INT |
| (Intercept) | -10,48 | 2,46 | -4,27 | 15 | 0,0007 | -15,72 | -5,24 | ICMS_pre S1 G INT |
| LesV | 0,08 | 0,25 | 0,34 | 15 | 0,7375 | -0,44 | 0,61 | ICMS_pre S1 G INT |
| LesVI | 0,21 | 0,11 | 1,83 | 15 | 0,0874 | -0,03 | 0,45 | ICMS_pre S1 G INT |
| (Intercept) | -28,10 | 4,61 | -6,09 | 15 | 2,06E-05 | -37,93 | -18,27 | ICMS_pre S1 IG INT |
| LesV | -0,14 | 0,46 | -0,31 | 15 | 0,7633 | -1,13 | 0,85 | ICMS_pre S1 IG INT |
| LesVI | 0,81 | 0,21 | 3,84 | 15 | **0,0016** | 0,36 | 1,26 | ICMS_pre S1 IG INT |
| (Intercept) | -3,05 | 0,95 | -3,21 | 15 | 0,0058 | -5,08 | -1,03 | ICMS_pre iS1 SG INT |
| LesV | 0,04 | 0,10 | 0,42 | 15 | 0,6791 | -0,16 | 0,24 | ICMS_pre iS1 SG INT |
| LesVI | 0,06 | 0,04 | 1,44 | 15 | 0,1702 | -0,03 | 0,16 | ICMS_pre iS1 SG INT |
| (Intercept) | -4,31 | 1,13 | -3,80 | 15 | 0,0017 | -6,73 | -1,89 | ICMS_pre iS1 G INT |
| LesV | 0,05 | 0,11 | 0,47 | 15 | 0,6451 | -0,19 | 0,30 | ICMS_pre iS1 G INT |
| LesVI | 0,09 | 0,05 | 1,79 | 15 | 0,0939 | -0,02 | 0,20 | ICMS_pre iS1 G INT |
| (Intercept) | -16,65 | 3,68 | -4,53 | 15 | 0,0004 | -24,49 | -8,81 | ICMS_pre iS1 IG INT |
| LesV | 0,37 | 0,37 | 1,01 | 15 | 0,3298 | -0,42 | 1,16 | ICMS_pre iS1 IG INT |
| LesVI | 0,35 | 0,17 | 2,05 | 15 | 0,0580 | -0,01 | 0,71 | ICMS_pre iS1 IG INT |
| (Intercept) | 8,32 | 1,48 | 5,62 | 14 | 0,0001 | 5,14 | 11,49 | ICMS_post AVR SG INT |
| LesV | 0,56 | 0,15 | 3,78 | 14 | **0,0020** | 0,24 | 0,88 | ICMS_post AVR SG INT |
| LesVI | 0,00 | 0,07 | 0,05 | 14 | 0,9582 | -0,15 | 0,15 | ICMS_post AVR SG INT |
| (Intercept) | 9,44 | 1,47 | 6,44 | 14 | 1,55E-05 | 6,30 | 12,59 | ICMS_post AVR G INT |
| LesV | 0,57 | 0,15 | 3,85 | 14 | **0,0018** | 0,25 | 0,88 | ICMS_post AVR G INT |
| LesVI | -0,05 | 0,07 | -0,74 | 14 | 0,4723 | -0,20 | 0,10 | ICMS_post AVR G INT |
| (Intercept) | 11,40 | 1,53 | 7,44 | 14 | 3,17E-06 | 8,11 | 14,69 | ICMS_post AVR IG INT |
| LesV | 0,62 | 0,15 | 4,03 | 14 | **0,0012** | 0,29 | 0,95 | ICMS_post AVR IG INT |
| LesVI | -0,14 | 0,07 | -1,94 | 14 | 0,0734 | -0,30 | 0,02 | ICMS_post AVR IG INT |
| (Intercept) | -1,21 | 0,44 | -2,74 | 14 | 0,0160 | -2,16 | -0,26 | ICMS_post S1 SG INT |
| LesV | 0,01 | 0,04 | 0,17 | 14 | 0,8712 | -0,09 | 0,10 | ICMS_post S1 SG INT |
| LesVI | 0,03 | 0,02 | 1,51 | 14 | 0,1525 | -0,01 | 0,08 | ICMS_post S1 SG INT |
| (Intercept) | -2,81 | 0,72 | -3,92 | 14 | 0,0016 | -4,35 | -1,27 | ICMS_post S1 G INT |
| LesV | 0,01 | 0,07 | 0,14 | 14 | 0,8929 | -0,14 | 0,16 | ICMS_post S1 G INT |
| LesVI | 0,07 | 0,03 | 2,11 | 14 | 0,0535 | 0,00 | 0,14 | ICMS_post S1 G INT |
| (Intercept) | -3,98 | 0,62 | -6,44 | 14 | 1,55E-05 | -5,31 | -2,65 | ICMS_post S1 IG INT |
| LesV | 0,03 | 0,06 | 0,55 | 14 | 0,5907 | -0,10 | 0,17 | ICMS_post S1 IG INT |
| LesVI | 0,11 | 0,03 | 3,71 | 14 | **0,0023** | 0,05 | 0,17 | ICMS_post S1 IG INT |
| (Intercept) | -3,22 | 0,81 | -3,96 | 14 | 0,0014 | -4,97 | -1,48 | ICMS_post iS1 SG INT |
| LesV | -0,17 | 0,08 | -2,05 | 14 | 0,0597 | -0,34 | 0,01 | ICMS_post iS1 SG INT |
| LesVI | 0,08 | 0,04 | 2,06 | 14 | 0,0583 | 0,00 | 0,16 | ICMS_post iS1 SG INT |
| (Intercept) | -4,14 | 0,93 | -4,46 | 14 | 0,0005 | -6,13 | -2,15 | ICMS_post iS1 G INT |
| LesV | -0,18 | 0,09 | -1,95 | 14 | 0,0717 | -0,38 | 0,02 | ICMS_post iS1 G INT |
| LesVI | 0,13 | 0,04 | 2,88 | 14 | **0,0122** | 0,03 | 0,22 | ICMS_post iS1 G INT |
| (Intercept) | -7,61 | 1,76 | -4,32 | 14 | 0,0007 | -11,39 | -3,83 | ICMS_post iS1 IG INT |
| LesV | -0,09 | 0,18 | -0,52 | 14 | 0,6096 | -0,47 | 0,29 | ICMS_post iS1 IG INT |
| LesVI | 0,18 | 0,08 | 2,21 | 14 | **0,0443** | 0,01 | 0,36 | ICMS_post iS1 IG INT |

**Suppl. Table 2a: Statistical analysis of layer specific lesion effects using a liner mixed-effects model with LesV (threshold 5%) and LesVI (threshold 15%) as categorical variables (yes,no); pre and post muscimol condition separately**

| **Predictor** | **Estimate** | **SE** | **tStat** | **DF** | **pValue** | **Lower** | **Upper** | **Dependent Variable** |
| --- | --- | --- | --- | --- | --- | --- | --- | --- |
| (Intercept) | 37,25 | 6,27 | 5,94 | 14 | 0,0000 | 23,80 | 50,71 | AcS_pre AVR BF INT |
| LesV_group | -2,97 | 9,00 | -0,33 | 14 | 0,7460 | -22,28 | 16,33 | AcS_pre AVR BF INT |
| LesVI_group | -8,09 | 9,00 | -0,90 | 14 | 0,3843 | -27,39 | 11,22 | AcS_pre AVR BF INT |
| (Intercept) | 16,52 | 0,48 | 34,12 | 12 | 2,55E-13 | 15,47 | 17,58 | AcS_pre AVR BF OL |
| LesV_group | 0,34 | 0,76 | 0,45 | 12 | 0,6609 | -1,31 | 1,99 | AcS_pre AVR BF OL |
| LesVI_group | 0,76 | 0,74 | 1,03 | 12 | 0,3231 | -0,85 | 2,38 | AcS_pre AVR BF OL |
| (Intercept) | -67,71 | 12,83 | -5,28 | 14 | 0,0001 | -95,23 | -40,20 | AcS_pre S1 BF INT |
| LesV_group | -12,77 | 18,41 | -0,69 | 14 | 0,4993 | -52,25 | 26,71 | AcS_pre S1 BF INT |
| LesVI_group | 23,57 | 18,41 | 1,28 | 14 | 0,2211 | -15,91 | 63,05 | AcS_pre S1 BF INT |
| (Intercept) | 18,89 | 0,53 | 35,47 | 14 | 4,12E-15 | 17,74 | 20,03 | AcS_pre S1 BF OL |
| LesV_group | 2,80 | 0,76 | 3,66 | 14 | **0,0026** | 1,16 | 4,44 | AcS_pre S1 BF OL |
| LesVI_group | -0,96 | 0,76 | -1,26 | 14 | 0,2273 | -2,60 | 0,67 | AcS_pre S1 BF OL |
| (Intercept) | -14,45 | 4,00 | -3,62 | 14 | 0,0028 | -23,02 | -5,88 | AcS_pre S1 nBF INT |
| LesV_group | -1,80 | 5,73 | -0,31 | 14 | 0,7586 | -14,09 | 10,50 | AcS_pre S1 nBF INT |
| LesVI_group | 2,68 | 5,73 | 0,47 | 14 | 0,6469 | -9,61 | 14,98 | AcS_pre S1 nBF INT |
| (Intercept) | 26,41 | 1,01 | 26,23 | 14 | 2,65E-13 | 24,25 | 28,57 | AcS_pre S1 nBF OL |
| LesV_group | 3,14 | 1,44 | 2,18 | 14 | **0,0471** | 0,05 | 6,24 | AcS_pre S1 nBF OL |
| LesVI_group | -4,39 | 1,44 | -3,04 | 14 | **0,0088** | -7,49 | -1,30 | AcS_pre S1 nBF OL |
| (Intercept) | -80,78 | 4,14 | -19,52 | 14 | 1,50E-11 | -89,66 | -71,90 | AcS_pre S1 rel_nBF INT |
| LesV_group | -4,59 | 5,94 | -0,77 | 14 | 0,4528 | -17,32 | 8,15 | AcS_pre S1 rel_nBF INT |
| LesVI_group | 11,84 | 5,94 | 1,99 | 14 | 0,0661 | -0,90 | 24,58 | AcS_pre S1 rel_nBF INT |
| (Intercept) | 7,52 | 0,87 | 8,69 | 14 | 5,16E-07 | 5,67 | 9,38 | AcS_pre S1 rel_nBF OL |
| LesV_group | 0,35 | 1,24 | 0,28 | 14 | 0,7844 | -2,32 | 3,01 | AcS_pre S1 rel_nBF OL |
| LesVI_group | -3,43 | 1,24 | -2,76 | 14 | **0,0153** | -6,09 | -0,77 | AcS_pre S1 rel_nBF OL |
| (Intercept) | -21,66 | 3,10 | -6,98 | 14 | 0,0000 | -28,31 | -15,00 | AcS_pre iS1 BF INT |
| LesV_group | 2,13 | 4,45 | 0,48 | 14 | 0,6393 | -7,42 | 11,68 | AcS_pre iS1 BF INT |
| LesVI_group | 14,83 | 4,45 | 3,33 | 14 | **0,0049** | 5,28 | 24,38 | AcS_pre iS1 BF INT |
| (Intercept) | 17,59 | 1,59 | 11,09 | 12 | 1,16E-07 | 14,13 | 21,05 | AcS_pre iS1 BF OL |
| LesV_group | -2,12 | 2,48 | -0,86 | 12 | 0,4091 | -7,51 | 3,28 | AcS_pre iS1 BF OL |
| LesVI_group | 3,24 | 2,43 | 1,33 | 12 | 0,2080 | -2,06 | 8,53 | AcS_pre iS1 BF OL |
| (Intercept) | -6,78 | 1,70 | -3,98 | 14 | 0,0014 | -10,44 | -3,13 | AcS_pre iS1 nBF INT |
| LesV_group | 0,69 | 2,44 | 0,28 | 14 | 0,7815 | -4,55 | 5,93 | AcS_pre iS1 nBF INT |
| LesVI_group | 2,04 | 2,44 | 0,83 | 14 | 0,4181 | -3,20 | 7,28 | AcS_pre iS1 nBF INT |
| (Intercept) | 22,24 | 2,44 | 9,11 | 13 | 5,25E-07 | 16,97 | 27,52 | AcS_pre iS1 nBF OL |
| LesV_group | -3,21 | 3,60 | -0,89 | 13 | 0,3881 | -10,99 | 4,56 | AcS_pre iS1 nBF OL |
| LesVI_group | 2,76 | 3,51 | 0,79 | 13 | 0,4456 | -4,82 | 10,35 | AcS_pre iS1 nBF OL |
| (Intercept) | -74,10 | 27,36 | -2,71 | 13 | 0,0179 | -133,21 | -14,99 | AcS_pre S2 BF INT |
| LesV_group | 13,47 | 40,33 | 0,33 | 13 | 0,7437 | -73,66 | 100,59 | AcS_pre S2 BF INT |
| LesVI_group | -38,23 | 39,36 | -0,97 | 13 | 0,3491 | -123,26 | 46,79 | AcS_pre S2 BF INT |
| (Intercept) | 20,31 | 1,79 | 11,32 | 11 | 2,11E-07 | 16,36 | 24,26 | AcS_pre S2 BF OL |
| LesV_group | -1,17 | 2,88 | -0,40 | 11 | 0,6933 | -7,51 | 5,17 | AcS_pre S2 BF OL |
| LesVI_group | 0,07 | 2,76 | 0,03 | 11 | 0,9798 | -6,01 | 6,15 | AcS_pre S2 BF OL |
| (Intercept) | 7,49 | 49,04 | 0,15 | 13 | 0,8809 | -98,44 | 113,43 | AcS_pre S2 rel_BF INT |
| LesV_group | -37,19 | 72,28 | -0,51 | 13 | 0,6155 | -193,33 | 118,95 | AcS_pre S2 rel_BF INT |
| LesVI_group | 172,09 | 70,53 | 2,44 | 13 | **0,0298** | 19,71 | 324,47 | AcS_pre S2 rel_BF INT |
| (Intercept) | -51,88 | 12,00 | -4,32 | 14 | 0,0007 | -77,61 | -26,14 | AcS_pre S3 BF INT |
| LesV_group | -11,67 | 17,22 | -0,68 | 14 | 0,5088 | -48,60 | 25,26 | AcS_pre S3 BF INT |
| LesVI_group | -7,55 | 17,22 | -0,44 | 14 | 0,6679 | -44,48 | 29,38 | AcS_pre S3 BF INT |
| (Intercept) | 20,31 | 1,79 | 11,32 | 11 | 2,11E-07 | 16,36 | 24,26 | AcS_pre S3 BF OL |
| LesV_group | -1,17 | 2,88 | -0,40 | 11 | 0,6933 | -7,51 | 5,17 | AcS_pre S3 BF OL |
| LesVI_group | 0,07 | 2,76 | 0,03 | 11 | 0,9798 | -6,01 | 6,15 | AcS_pre S3 BF OL |
| (Intercept) | -15,00 | 28,53 | -0,53 | 14 | 0,6073 | -76,19 | 46,20 | AcS_pre S3 rel_BF INT |
| LesV_group | 17,03 | 40,94 | 0,42 | 14 | 0,6838 | -70,78 | 104,83 | AcS_pre S3 rel_BF INT |
| LesVI_group | 65,87 | 40,94 | 1,61 | 14 | 0,1299 | -21,93 | 153,68 | AcS_pre S3 rel_BF INT |
| (Intercept) | 8,58 | 1,95 | 4,41 | 13 | 0,0007 | 4,37 | 12,79 | AcS_post AVR BF INT |
| LesV_group | -2,57 | 2,87 | -0,89 | 13 | 0,3872 | -8,77 | 3,63 | AcS_post AVR BF INT |
| LesVI_group | -0,68 | 2,80 | -0,24 | 13 | 0,8107 | -6,73 | 5,37 | AcS_post AVR BF INT |
| (Intercept) | 17,08 | 0,93 | 18,31 | 13 | 1,15E-10 | 15,06 | 19,09 | AcS_post AVR BF OL |
| LesV_group | 1,46 | 1,37 | 1,06 | 13 | 0,3079 | -1,51 | 4,43 | AcS_post AVR BF OL |
| LesVI_group | -0,44 | 1,34 | -0,33 | 13 | 0,7463 | -3,34 | 2,45 | AcS_post AVR BF OL |
| (Intercept) | -16,44 | 4,21 | -3,91 | 13 | 0,0018 | -25,54 | -7,35 | AcS_post S1 BF INT |
| LesV_group | 2,68 | 6,20 | 0,43 | 13 | 0,6728 | -10,72 | 16,08 | AcS_post S1 BF INT |
| LesVI_group | 4,33 | 6,05 | 0,71 | 13 | 0,4875 | -8,75 | 17,41 | AcS_post S1 BF INT |
| (Intercept) | 18,37 | 0,68 | 27,09 | 13 | 8,02E-13 | 16,91 | 19,84 | AcS_post S1 BF OL |
| LesV_group | 2,40 | 1,00 | 2,40 | 13 | **0,0319** | 0,24 | 4,56 | AcS_post S1 BF OL |
| LesVI_group | -0,93 | 0,98 | -0,95 | 13 | 0,3588 | -3,03 | 1,18 | AcS_post S1 BF OL |
| (Intercept) | -3,36 | 1,77 | -1,89 | 12 | 0,0827 | -7,22 | 0,51 | AcS_post S1 nBF INT |
| LesV_group | -0,22 | 2,91 | -0,07 | 12 | 0,9418 | -6,56 | 6,13 | AcS_post S1 nBF INT |
| LesVI_group | 0,05 | 2,80 | 0,02 | 12 | 0,9858 | -6,05 | 6,16 | AcS_post S1 nBF INT |
| (Intercept) | 23,10 | 1,50 | 15,37 | 9 | 9,10E-08 | 19,70 | 26,50 | AcS_post S1 nBF OL |
| LesV_group | 2,75 | 2,57 | 1,07 | 9 | 0,3117 | -3,05 | 8,55 | AcS_post S1 nBF OL |
| LesVI_group | 0,65 | 2,25 | 0,29 | 9 | 0,7796 | -4,45 | 5,75 | AcS_post S1 nBF OL |
| (Intercept) | -81,70 | 5,26 | -15,52 | 12 | 2,63E-09 | -93,17 | -70,23 | AcS_post S1 rel_nBF INT |
| LesV_group | -3,80 | 8,65 | -0,44 | 12 | 0,6685 | -22,64 | 15,05 | AcS_post S1 rel_nBF INT |
| LesVI_group | 12,65 | 8,32 | 1,52 | 12 | 0,1544 | -5,48 | 30,79 | AcS_post S1 rel_nBF INT |
| (Intercept) | 5,90 | 1,29 | 4,59 | 9 | 0,0013 | 2,99 | 8,81 | AcS_post S1 rel_nBF OL |
| LesV_group | 2,42 | 2,20 | 1,10 | 9 | 0,2995 | -2,55 | 7,38 | AcS_post S1 rel_nBF OL |
| LesVI_group | -0,15 | 1,93 | -0,08 | 9 | 0,9397 | -4,51 | 4,21 | AcS_post S1 rel_nBF OL |
| (Intercept) | -75,41 | 3,55 | -21,23 | 13 | 1,78E-11 | -83,08 | -67,74 | AcS_post S1 rel_preBF INT |
| LesV_group | -10,68 | 5,23 | -2,04 | 13 | 0,0621 | -21,99 | 0,63 | AcS_post S1 rel_preBF INT |
| LesVI_group | -1,65 | 5,11 | -0,32 | 13 | 0,7518 | -12,69 | 9,39 | AcS_post S1 rel_preBF INT |
| (Intercept) | -6,16 | 1,72 | -3,58 | 13 | 0,0034 | -9,88 | -2,44 | AcS_post iS1 BF INT |
| LesV_group | 0,98 | 2,54 | 0,39 | 13 | 0,7056 | -4,50 | 6,46 | AcS_post iS1 BF INT |
| LesVI_group | 3,95 | 2,48 | 1,59 | 13 | 0,1347 | -1,40 | 9,30 | AcS_post iS1 BF INT |
| (Intercept) | 17,59 | 3,79 | 4,64 | 10 | 0,0009 | 9,13 | 26,04 | AcS_post iS1 BF OL |
| LesV_group | -3,53 | 5,64 | -0,63 | 10 | 0,5452 | -16,10 | 9,04 | AcS_post iS1 BF OL |
| LesVI_group | 7,43 | 5,51 | 1,35 | 10 | 0,2070 | -4,84 | 19,70 | AcS_post iS1 BF OL |
| (Intercept) | -1,65 | 0,64 | -2,59 | 12 | 0,0237 | -3,04 | -0,26 | AcS_post iS1 nBF INT |
| LesV_group | -0,15 | 1,05 | -0,15 | 12 | 0,8849 | -2,44 | 2,13 | AcS_post iS1 nBF INT |
| LesVI_group | 1,01 | 1,01 | 1,01 | 12 | 0,3339 | -1,18 | 3,21 | AcS_post iS1 nBF INT |
| (Intercept) | 9,84 | 1,65 | 5,95 | 15 | 0,0000 | 6,32 | 13,36 | ICMS_pre AVR SG INT |
| LesV_group | 0,70 | 2,23 | 0,31 | 15 | 0,7582 | -4,05 | 5,45 | ICMS_pre AVR SG INT |
| LesVI_group | 1,29 | 2,23 | 0,58 | 15 | 0,5718 | -3,46 | 6,04 | ICMS_pre AVR SG INT |
| (Intercept) | 17,10 | 3,26 | 5,24 | 15 | 9,98E-05 | 10,14 | 24,05 | ICMS_pre AVR G INT |
| LesV_group | -0,35 | 4,40 | -0,08 | 15 | 0,9378 | -9,73 | 9,03 | ICMS_pre AVR G INT |
| LesVI_group | -3,47 | 4,40 | -0,79 | 15 | 0,4427 | -12,85 | 5,91 | ICMS_pre AVR G INT |
| (Intercept) | 27,60 | 2,97 | 9,30 | 15 | 1,29E-07 | 21,27 | 33,92 | ICMS_pre AVR IG INT |
| LesV_group | 0,16 | 4,00 | 0,04 | 15 | 0,9696 | -8,37 | 8,68 | ICMS_pre AVR IG INT |
| LesVI_group | -13,36 | 4,00 | -3,34 | 15 | **0,0045** | -21,89 | -4,83 | ICMS_pre AVR IG INT |
| (Intercept) | -2,18 | 0,50 | -4,37 | 15 | 0,0005 | -3,25 | -1,12 | ICMS_pre S1 SG INT |
| LesV_group | 0,56 | 0,67 | 0,83 | 15 | 0,4208 | -0,88 | 1,99 | ICMS_pre S1 SG INT |
| LesVI_group | 1,57 | 0,67 | 2,33 | 15 | **0,0341** | 0,13 | 3,01 | ICMS_pre S1 SG INT |
| (Intercept) | -8,03 | 2,41 | -3,33 | 15 | 0,0046 | -13,17 | -2,89 | ICMS_pre S1 G INT |
| LesV_group | -1,82 | 3,25 | -0,56 | 15 | 0,5829 | -8,75 | 5,10 | ICMS_pre S1 G INT |
| LesVI_group | 4,78 | 3,25 | 1,47 | 15 | 0,1619 | -2,15 | 11,71 | ICMS_pre S1 G INT |
| (Intercept) | -24,14 | 4,30 | -5,61 | 15 | 0,0000 | -33,31 | -14,96 | ICMS_pre S1 IG INT |
| LesV_group | -3,07 | 5,80 | -0,53 | 15 | 0,6051 | -15,44 | 9,31 | ICMS_pre S1 IG INT |
| LesVI_group | 22,56 | 5,80 | 3,89 | 15 | **0,0015** | 10,19 | 34,94 | ICMS_pre S1 IG INT |
| (Intercept) | -3,52 | 0,77 | -4,56 | 15 | 3,79E-04 | -5,17 | -1,87 | ICMS_pre iS1 SG INT |
| LesV_group | 0,96 | 1,04 | 0,92 | 15 | 0,3736 | -1,27 | 3,18 | ICMS_pre iS1 SG INT |
| LesVI_group | 2,54 | 1,04 | 2,44 | 15 | **0,0278** | 0,32 | 4,76 | ICMS_pre iS1 SG INT |
| (Intercept) | -4,91 | 0,88 | -5,60 | 15 | 0,0001 | -6,78 | -3,04 | ICMS_pre iS1 G INT |
| LesV_group | 1,54 | 1,18 | 1,30 | 15 | 0,2116 | -0,98 | 4,07 | ICMS_pre iS1 G INT |
| LesVI_group | 3,44 | 1,18 | 2,91 | 15 | **0,0108** | 0,92 | 5,97 | ICMS_pre iS1 G INT |
| (Intercept) | -15,74 | 3,47 | -4,54 | 15 | 0,0004 | -23,13 | -8,35 | ICMS_pre iS1 IG INT |
| LesV_group | 4,08 | 4,67 | 0,87 | 15 | 0,3966 | -5,88 | 14,04 | ICMS_pre iS1 IG INT |
| LesVI_group | 9,68 | 4,67 | 2,07 | 15 | 0,0560 | -0,28 | 19,64 | ICMS_pre iS1 IG INT |
| (Intercept) | 6,45 | 1,15 | 5,61 | 14 | 0,0001 | 3,99 | 8,91 | ICMS_post AVR SG INT |
| LesV_group | 7,17 | 1,58 | 4,53 | 14 | **0,0005** | 3,78 | 10,56 | ICMS_post AVR SG INT |
| LesVI_group | 1,52 | 1,56 | 0,98 | 14 | 0,3450 | -1,82 | 4,87 | ICMS_post AVR SG INT |
| (Intercept) | 7,10 | 1,04 | 6,85 | 14 | 0,0000 | 4,88 | 9,33 | ICMS_post AVR G INT |
| LesV_group | 8,02 | 1,43 | 5,62 | 14 | **0,0001** | 4,96 | 11,08 | ICMS_post AVR G INT |
| LesVI_group | -0,06 | 1,41 | -0,05 | 14 | 0,9639 | -3,08 | 2,95 | ICMS_post AVR G INT |
| (Intercept) | 8,45 | 1,16 | 7,25 | 14 | 4,22E-06 | 5,95 | 10,94 | ICMS_post AVR IG INT |
| LesV_group | 8,86 | 1,60 | 5,52 | 14 | **0,0001** | 5,42 | 12,30 | ICMS_post AVR IG INT |
| LesVI_group | -2,23 | 1,58 | -1,41 | 14 | 0,1802 | -5,62 | 1,16 | ICMS_post AVR IG INT |
| (Intercept) | -1,23 | 0,36 | -3,41 | 14 | 4,21E-03 | -2,00 | -0,46 | ICMS_post S1 SG INT |
| LesV_group | -0,14 | 0,50 | -0,28 | 14 | **0,7801** | -1,20 | 0,92 | ICMS_post S1 SG INT |
| LesVI_group | 1,26 | 0,49 | 2,57 | 14 | **0,0221** | 0,21 | 2,31 | ICMS_post S1 SG INT |
| (Intercept) | -2,52 | 0,60 | -4,20 | 14 | 0,0009 | -3,81 | -1,23 | ICMS_post S1 G INT |
| LesV_group | -0,55 | 0,83 | -0,67 | 14 | 0,5136 | -2,33 | 1,22 | ICMS_post S1 G INT |
| LesVI_group | 2,36 | 0,82 | 2,89 | 14 | **0,0118** | 0,61 | 4,11 | ICMS_post S1 G INT |
| (Intercept) | -3,54 | 0,52 | -6,82 | 14 | 0,0000 | -4,65 | -2,42 | ICMS_post S1 IG INT |
| LesV_group | -0,15 | 0,71 | -0,21 | 14 | 0,8391 | -1,68 | 1,38 | ICMS_post S1 IG INT |
| LesVI_group | 3,15 | 0,70 | 4,47 | 14 | **0,0005** | 1,64 | 4,66 | ICMS_post S1 IG INT |
| (Intercept) | -2,30 | 0,77 | -3,00 | 14 | 9,58E-03 | -3,95 | -0,66 | ICMS_post iS1 SG INT |
| LesV_group | -2,01 | 1,06 | -1,90 | 14 | 0,0781 | -4,28 | 0,26 | ICMS_post iS1 SG INT |
| LesVI_group | 1,48 | 1,04 | 1,42 | 14 | **0,1776** | -0,76 | 3,72 | ICMS_post iS1 SG INT |
| (Intercept) | -3,08 | 0,92 | -3,35 | 14 | 0,0048 | -5,05 | -1,11 | ICMS_post iS1 G INT |
| LesV_group | -1,83 | 1,27 | -1,45 | 14 | 0,1700 | -4,55 | 0,88 | ICMS_post iS1 G INT |
| LesVI_group | 2,58 | 1,25 | 2,07 | 14 | 0,0579 | -0,10 | 5,26 | ICMS_post iS1 G INT |
| (Intercept) | -5,58 | 1,71 | -3,27 | 14 | 0,0055 | -9,24 | -1,93 | ICMS_post iS1 IG INT |
| LesV_group | -1,70 | 2,35 | -0,72 | 14 | 0,4811 | -6,73 | 3,34 | ICMS_post iS1 IG INT |
| LesVI_group | 3,14 | 2,31 | 1,36 | 14 | 0,1963 | -1,82 | 8,10 | ICMS_post iS1 IG INT |

**Suppl. Table 2b: Statistical analysis of layer specific lesion effects using a liner mixed-effects model with LesV (threshold 5%) and LesVI (threshold 10%) as categorical variables (yes,no); pre and post muscimol condition separately**

| **Predictor** | **Estimate** | **SE** | **tStat** | | **DF** | **pValue** | **Lower** | **Upper** | **Dependent Variable** |
| --- | --- | --- | --- | --- | --- | --- | --- | --- | --- |
| (Intercept) | 39,29 | 6,66 | | 5,90 | 14 | 0,0000 | 24,99 | 53,58 | AcS_pre AVR BF INT |
| Les5_group | -2,91 | 8,46 | | -0,34 | 14 | 0,7360 | -21,04 | 15,23 | AcS_pre AVR BF INT |
| Les6_group | -10,54 | 8,71 | | -1,21 | 14 | 0,2464 | -29,21 | 8,14 | AcS_pre AVR BF INT |
| (Intercept) | 16,28 | 0,50 | | 32,60 | 12 | 4,39E-13 | 15,20 | 17,37 | AcS_pre AVR BF OL |
| Les5_group | 0,29 | 0,68 | | 0,43 | 12 | 0,6771 | -1,19 | 1,78 | AcS_pre AVR BF OL |
| Les6_group | 1,11 | 0,68 | | 1,63 | 12 | 0,1297 | -0,38 | 2,59 | AcS_pre AVR BF OL |
| (Intercept) | -71,07 | 13,73 | | -5,18 | 14 | 0,0001 | -100,53 | -41,62 | AcS_pre S1 BF INT |
| Les5_group | -11,13 | 17,42 | | -0,64 | 14 | 0,5334 | -48,50 | 26,24 | AcS_pre S1 BF INT |
| Les6_group | 25,58 | 17,94 | | 1,43 | 14 | 0,1759 | -12,90 | 64,06 | AcS_pre S1 BF INT |
| (Intercept) | 19,04 | 0,57 | | 33,54 | 14 | 8,92E-15 | 17,82 | 20,26 | AcS_pre S1 BF OL |
| Les5_group | 2,74 | 0,72 | | 3,81 | 14 | **0,0019** | 1,20 | 4,29 | AcS_pre S1 BF OL |
| Les6_group | -1,09 | 0,74 | | -1,46 | 14 | 0,1656 | -2,68 | 0,51 | AcS_pre S1 BF OL |
| (Intercept) | -15,92 | 4,24 | | -3,75 | 14 | 0,0021 | -25,03 | -6,82 | AcS_pre S1 nBF INT |
| Les5_group | -2,39 | 5,39 | | -0,44 | 14 | 0,6640 | -13,94 | 9,16 | AcS_pre S1 nBF INT |
| Les6_group | 5,10 | 5,55 | | 0,92 | 14 | 0,3737 | -6,80 | 16,99 | AcS_pre S1 nBF INT |
| (Intercept) | 26,36 | 1,20 | | 22,04 | 14 | 2,86E-12 | 23,79 | 28,92 | AcS_pre S1 nBF OL |
| Les5_group | 2,36 | 1,52 | | 1,55 | 14 | 0,1428 | -0,90 | 5,61 | AcS_pre S1 nBF OL |
| Les6_group | -3,41 | 1,56 | | -2,19 | 14 | **0,0463** | -6,77 | -0,06 | AcS_pre S1 nBF OL |
| (Intercept) | -79,73 | 4,78 | | -16,68 | 14 | 1,24E-10 | -89,99 | -69,48 | AcS_pre S1 rel_nBF INT |
| Les5_group | -1,81 | 6,07 | | -0,30 | 14 | 0,7697 | -14,82 | 11,20 | AcS_pre S1 rel_nBF INT |
| Les6_group | 7,38 | 6,25 | | 1,18 | 14 | 0,2570 | -6,02 | 20,78 | AcS_pre S1 rel_nBF INT |
| (Intercept) | 7,31 | 1,04 | | 7,03 | 14 | 5,97E-06 | 5,08 | 9,55 | AcS_pre S1 rel_nBF OL |
| Les5_group | -0,39 | 1,32 | | -0,29 | 14 | 0,7724 | -3,22 | 2,44 | AcS_pre S1 rel_nBF OL |
| Les6_group | -2,33 | 1,36 | | -1,71 | 14 | 0,1087 | -5,25 | 0,59 | AcS_pre S1 rel_nBF OL |
| (Intercept) | -24,41 | 2,87 | | -8,51 | 14 | 0,0000 | -30,56 | -18,26 | AcS_pre iS1 BF INT |
| Les5_group | 2,71 | 3,64 | | 0,74 | 14 | 0,4686 | -5,09 | 10,52 | AcS_pre iS1 BF INT |
| Les6_group | 17,37 | 3,75 | | 4,63 | 14 | **0,0004** | 9,33 | 25,40 | AcS_pre iS1 BF INT |
| (Intercept) | 16,12 | 1,45 | | 11,08 | 12 | 1,17E-07 | 12,95 | 19,29 | AcS_pre iS1 BF OL |
| Les5_group | -2,73 | 1,99 | | -1,37 | 12 | 0,1946 | -7,05 | 1,60 | AcS_pre iS1 BF OL |
| Les6_group | 5,73 | 1,99 | | 2,89 | 12 | **0,0137** | 1,40 | 10,05 | AcS_pre iS1 BF OL |
| (Intercept) | -7,63 | 1,78 | | -4,30 | 14 | 0,0007 | -11,44 | -3,82 | AcS_pre iS1 nBF INT |
| Les5_group | 0,44 | 2,25 | | 0,19 | 14 | 0,8487 | -4,39 | 5,27 | AcS_pre iS1 nBF INT |
| Les6_group | 3,32 | 2,32 | | 1,43 | 14 | 0,1746 | -1,66 | 8,30 | AcS_pre iS1 nBF INT |
| (Intercept) | 22,06 | 2,65 | | 8,33 | 13 | 1,43E-06 | 16,34 | 27,78 | AcS_pre iS1 nBF OL |
| Les5_group | -2,88 | 3,47 | | -0,83 | 13 | 0,4218 | -10,36 | 4,61 | AcS_pre iS1 nBF OL |
| Les6_group | 2,58 | 3,47 | | 0,74 | 13 | 0,4708 | -4,91 | 10,06 | AcS_pre iS1 nBF OL |
| (Intercept) | -81,84 | 30,33 | | -2,70 | 13 | 0,0182 | -147,37 | -16,32 | AcS_pre S2 BF INT |
| Les5_group | 1,93 | 39,71 | | 0,05 | 13 | 0,9619 | -83,85 | 87,72 | AcS_pre S2 BF INT |
| Les6_group | -15,10 | 39,71 | | -0,38 | 13 | 0,7099 | -100,88 | 70,69 | AcS_pre S2 BF INT |
| (Intercept) | 18,59 | 1,78 | | 10,43 | 11 | 4,87E-07 | 14,66 | 22,51 | AcS_pre S2 BF OL |
| Les5_group | -2,53 | 2,52 | | -1,00 | 11 | 0,3374 | -8,08 | 3,02 | AcS_pre S2 BF OL |
| Les6_group | 3,93 | 2,44 | | 1,61 | 11 | 0,1361 | -1,45 | 9,30 | AcS_pre S2 BF OL |
| (Intercept) | 14,40 | 57,47 | | 0,25 | 13 | 0,8060 | -109,76 | 138,56 | AcS_pre S2 rel_BF INT |
| Les5_group | -3,90 | 75,25 | | -0,05 | 13 | 0,9595 | -166,47 | 158,67 | AcS_pre S2 rel_BF INT |
| Les6_group | 123,85 | 75,25 | | 1,65 | 13 | 0,1237 | -38,72 | 286,41 | AcS_pre S2 rel_BF INT |
| (Intercept) | -47,79 | 12,78 | | -3,74 | 14 | 0,0022 | -75,21 | -20,37 | AcS_pre S3 BF INT |
| Les5_group | -10,05 | 16,22 | | -0,62 | 14 | 0,5454 | -44,84 | 24,74 | AcS_pre S3 BF INT |
| Les6_group | -14,20 | 16,70 | | -0,85 | 14 | 0,4095 | -50,03 | 21,62 | AcS_pre S3 BF INT |
| (Intercept) | 18,59 | 1,78 | | 10,43 | 11 | 4,87E-07 | 14,66 | 22,51 | AcS_pre S3 BF OL |
| Les5_group | -2,53 | 2,52 | | -1,00 | 11 | 0,3374 | -8,08 | 3,02 | AcS_pre S3 BF OL |
| Les6_group | 3,93 | 2,44 | | 1,61 | 11 | 0,1361 | -1,45 | 9,30 | AcS_pre S3 BF OL |
| (Intercept) | -27,64 | 29,80 | | -0,93 | 14 | 0,3694 | -91,55 | 36,28 | AcS_pre S3 rel_BF INT |
| Les5_group | 19,29 | 37,81 | | 0,51 | 14 | 0,6178 | -61,80 | 100,38 | AcS_pre S3 rel_BF INT |
| Les6_group | 77,97 | 38,94 | | 2,00 | 14 | 0,0650 | -5,54 | 161,49 | AcS_pre S3 rel_BF INT |
| (Intercept) | 9,21 | 2,08 | | 4,42 | 13 | 0,0007 | 4,71 | 13,71 | AcS_post AVR BF INT |
| Les5_group | -2,26 | 2,73 | | -0,83 | 13 | 0,4218 | -8,15 | 3,63 | AcS_post AVR BF INT |
| Les6_group | -1,81 | 2,73 | | -0,66 | 13 | 0,5186 | -7,70 | 4,08 | AcS_post AVR BF INT |
| (Intercept) | 16,80 | 1,01 | | 16,60 | 13 | 3,92E-10 | 14,61 | 18,99 | AcS_post AVR BF OL |
| Les5_group | 1,20 | 1,32 | | 0,91 | 13 | 0,3815 | -1,66 | 4,06 | AcS_post AVR BF OL |
| Les6_group | 0,20 | 1,32 | | 0,15 | 13 | 0,8823 | -2,66 | 3,06 | AcS_post AVR BF OL |
| (Intercept) | -17,83 | 4,47 | | -3,99 | 13 | 0,0015 | -27,49 | -8,18 | AcS_post S1 BF INT |
| Les5_group | 2,47 | 5,85 | | 0,42 | 13 | 0,6794 | -10,17 | 15,12 | AcS_post S1 BF INT |
| Les6_group | 6,24 | 5,85 | | 1,07 | 13 | 0,3055 | -6,40 | 18,89 | AcS_post S1 BF INT |
| (Intercept) | 18,30 | 0,75 | | 24,55 | 13 | 2,82E-12 | 16,69 | 19,91 | AcS_post S1 BF OL |
| Les5_group | 2,20 | 0,98 | | 2,25 | 13 | **0,0421** | 0,09 | 4,31 | AcS_post S1 BF OL |
| Les6_group | -0,60 | 0,98 | | -0,61 | 13 | 0,5493 | -2,71 | 1,51 | AcS_post S1 BF OL |
| (Intercept) | -3,80 | 1,93 | | -1,97 | 12 | 0,0729 | -8,02 | 0,41 | AcS_post S1 nBF INT |
| Les5_group | -0,65 | 2,73 | | -0,24 | 12 | 0,8152 | -6,61 | 5,31 | AcS_post S1 nBF INT |
| Les6_group | 0,93 | 2,73 | | 0,34 | 12 | 0,7386 | -5,02 | 6,89 | AcS_post S1 nBF INT |
| (Intercept) | 22,50 | 1,65 | | 13,64 | 9 | 2,58E-07 | 18,77 | 26,23 | AcS_post S1 nBF OL |
| Les5_group | 2,40 | 2,41 | | 1,00 | 9 | 0,3454 | -3,05 | 7,85 | AcS_post S1 nBF OL |
| Les6_group | 1,60 | 2,21 | | 0,72 | 9 | 0,4882 | -3,41 | 6,61 | AcS_post S1 nBF OL |
| (Intercept) | -80,89 | 6,00 | | -13,49 | 12 | 1,30E-08 | -93,96 | -67,82 | AcS_post S1 rel_nBF INT |
| Les5_group | -0,45 | 8,48 | | -0,05 | 12 | 0,9584 | -18,93 | 18,03 | AcS_post S1 rel_nBF INT |
| Les6_group | 8,49 | 8,48 | | 1,00 | 12 | 0,3365 | -9,99 | 26,97 | AcS_post S1 rel_nBF INT |
| (Intercept) | 5,75 | 1,44 | | 4,00 | 9 | 0,0031 | 2,50 | 9,00 | AcS_post S1 rel_nBF OL |
| Les5_group | 2,27 | 2,10 | | 1,08 | 9 | 0,3083 | -2,48 | 7,01 | AcS_post S1 rel_nBF OL |
| Les6_group | 0,15 | 1,93 | | 0,08 | 9 | 0,9397 | -4,21 | 4,51 | AcS_post S1 rel_nBF OL |
| (Intercept) | -74,09 | 3,78 | | -19,60 | 13 | 4,90E-11 | -82,25 | -65,92 | AcS_post S1 rel_preBF INT |
| Les5_group | -10,07 | 4,95 | | -2,04 | 13 | 0,0627 | -20,77 | 0,62 | AcS_post S1 rel_preBF INT |
| Les6_group | -3,97 | 4,95 | | -0,80 | 13 | 0,4371 | -14,66 | 6,72 | AcS_post S1 rel_preBF INT |
| (Intercept) | -7,18 | 1,74 | | -4,12 | 13 | 0,0012 | -10,95 | -3,42 | AcS_post iS1 BF INT |
| Les5_group | 0,96 | 2,28 | | 0,42 | 13 | 0,6818 | -3,97 | 5,88 | AcS_post iS1 BF INT |
| Les6_group | 5,21 | 2,28 | | 2,28 | 13 | **0,0399** | 0,28 | 10,13 | AcS_post iS1 BF INT |
| (Intercept) | 17,59 | 3,79 | | 4,64 | 10 | 0,0009 | 9,13 | 26,04 | AcS_post iS1 BF OL |
| Les5_group | -3,53 | 5,64 | | -0,63 | 10 | 0,5452 | -16,10 | 9,04 | AcS_post iS1 BF OL |
| Les6_group | 7,43 | 5,51 | | 1,35 | 10 | 0,2070 | -4,84 | 19,70 | AcS_post iS1 BF OL |
| (Intercept) | -1,95 | 0,67 | | -2,90 | 12 | 0,0134 | -3,42 | -0,48 | AcS_post iS1 nBF INT |
| Les5_group | -0,25 | 0,95 | | -0,27 | 12 | 0,7953 | -2,33 | 1,82 | AcS_post iS1 nBF INT |
| Les6_group | 1,41 | 0,95 | | 1,48 | 12 | 0,1638 | -0,66 | 3,49 | AcS_post iS1 nBF INT |
| (Intercept) | 9,42 | 1,75 | | 5,37 | 15 | 0,0001 | 5,68 | 13,16 | ICMS_pre AVR SG INT |
| Les5_group | 0,68 | 2,14 | | 0,32 | 15 | 0,7541 | -3,87 | 5,23 | ICMS_pre AVR SG INT |
| Les6_group | 1,87 | 2,18 | | 0,86 | 15 | 0,4029 | -2,77 | 6,51 | ICMS_pre AVR SG INT |
| (Intercept) | 17,38 | 3,51 | | 4,96 | 15 | 1,71E-04 | 9,91 | 24,86 | ICMS_pre AVR G INT |
| Les5_group | -0,73 | 4,27 | | -0,17 | 15 | 0,8672 | -9,82 | 8,37 | ICMS_pre AVR G INT |
| Les6_group | -3,35 | 4,35 | | -0,77 | 15 | 0,4529 | -12,62 | 5,92 | ICMS_pre AVR G INT |
| (Intercept) | 29,93 | 2,81 | | 10,64 | 15 | 2,20E-08 | 23,93 | 35,93 | ICMS_pre AVR IG INT |
| Les5_group | -0,68 | 3,42 | | -0,20 | 15 | 0,8448 | -7,98 | 6,62 | ICMS_pre AVR IG INT |
| Les6_group | -15,36 | 3,49 | | -4,40 | 15 | **0,0005** | -22,80 | -7,92 | ICMS_pre AVR IG INT |
| (Intercept) | -1,86 | 0,60 | | -3,10 | 15 | 0,0073 | -3,14 | -0,58 | ICMS_pre S1 SG INT |
| Les5_group | 0,95 | 0,73 | | 1,31 | 15 | 0,2115 | -0,60 | 2,51 | ICMS_pre S1 SG INT |
| Les6_group | 0,61 | 0,74 | | 0,82 | 15 | 0,4246 | -0,98 | 2,20 | ICMS_pre S1 SG INT |
| (Intercept) | -8,32 | 2,61 | | -3,19 | 15 | 0,0061 | -13,88 | -2,76 | ICMS_pre S1 G INT |
| Les5_group | -1,25 | 3,17 | | -0,39 | 15 | 0,6985 | -8,02 | 5,51 | ICMS_pre S1 G INT |
| Les6_group | 4,41 | 3,23 | | 1,36 | 15 | 0,1930 | -2,49 | 11,30 | ICMS_pre S1 G INT |
| (Intercept) | -27,09 | 4,30 | | -6,29 | 15 | 0,0000 | -36,27 | -17,92 | ICMS_pre S1 IG INT |
| Les5_group | -1,16 | 5,24 | | -0,22 | 15 | 0,8278 | -12,32 | 10,01 | ICMS_pre S1 IG INT |
| Les6_group | 23,97 | 5,34 | | 4,49 | 15 | **0,0004** | 12,59 | 35,35 | ICMS_pre S1 IG INT |
| (Intercept) | -3,51 | 0,88 | | -3,99 | 15 | 1,18E-03 | -5,38 | -1,63 | ICMS_pre iS1 SG INT |
| Les5_group | 1,35 | 1,07 | | 1,26 | 15 | 0,2274 | -0,93 | 3,62 | ICMS_pre iS1 SG INT |
| Les6_group | 2,00 | 1,09 | | 1,83 | 15 | **0,0865** | -0,32 | 4,32 | ICMS_pre iS1 SG INT |
| (Intercept) | -4,81 | 1,03 | | -4,65 | 15 | 0,0003 | -7,01 | -2,60 | ICMS_pre iS1 G INT |
| Les5_group | 2,11 | 1,26 | | 1,68 | 15 | 0,1141 | -0,57 | 4,80 | ICMS_pre iS1 G INT |
| Les6_group | 2,55 | 1,28 | | 1,98 | 15 | **0,0659** | -0,19 | 5,28 | ICMS_pre iS1 G INT |
| (Intercept) | -17,56 | 3,53 | | -4,98 | 15 | 0,0002 | -25,09 | -10,04 | ICMS_pre iS1 IG INT |
| Les5_group | 4,62 | 4,30 | | 1,08 | 15 | 0,2991 | -4,54 | 13,77 | ICMS_pre iS1 IG INT |
| Les6_group | 11,39 | 4,38 | | 2,60 | 15 | **0,0200** | 2,06 | 20,72 | ICMS_pre iS1 IG INT |
| (Intercept) | 6,00 | 1,20 | | 5,00 | 14 | 0,0002 | 3,43 | 8,57 | ICMS_post AVR SG INT |
| Les5_group | 7,19 | 1,50 | | 4,81 | 14 | **0,0003** | 3,98 | 10,40 | ICMS_post AVR SG INT |
| Les6_group | 2,11 | 1,50 | | 1,41 | 14 | 0,1794 | -1,09 | 5,32 | ICMS_post AVR SG INT |
| (Intercept) | 6,76 | 1,11 | | 6,11 | 14 | 0,0000 | 4,38 | 9,13 | ICMS_post AVR G INT |
| Les5_group | 7,87 | 1,38 | | 5,70 | 14 | **0,0001** | 4,91 | 10,83 | ICMS_post AVR G INT |
| Les6_group | 0,63 | 1,38 | | 0,46 | 14 | 0,6532 | -2,33 | 3,60 | ICMS_post AVR G INT |
| (Intercept) | 8,29 | 1,29 | | 6,42 | 14 | 1,61E-05 | 5,52 | 11,06 | ICMS_post AVR IG INT |
| Les5_group | 8,47 | 1,61 | | 5,26 | 14 | **0,0001** | 5,01 | 11,92 | ICMS_post AVR IG INT |
| Les6_group | -1,47 | 1,61 | | -0,91 | 14 | 0,3784 | -4,92 | 1,99 | ICMS_post AVR IG INT |
| (Intercept) | -0,92 | 0,45 | | -2,05 | 14 | 6,00E-02 | -1,88 | 0,04 | ICMS_post S1 SG INT |
| Les5_group | 0,17 | 0,56 | | 0,30 | 14 | 0,7652 | -1,03 | 1,37 | ICMS_post S1 SG INT |
| Les6_group | 0,39 | 0,56 | | 0,69 | 14 | **0,5001** | -0,81 | 1,59 | ICMS_post S1 SG INT |
| (Intercept) | -2,39 | 0,72 | | -3,31 | 14 | 0,0051 | -3,95 | -0,84 | ICMS_post S1 G INT |
| Les5_group | -0,16 | 0,90 | | -0,18 | 14 | 0,8599 | -2,10 | 1,77 | ICMS_post S1 G INT |
| Les6_group | 1,63 | 0,90 | | 1,81 | 14 | 0,0922 | -0,30 | 3,56 | ICMS_post S1 G INT |
| (Intercept) | -3,60 | 0,64 | | -5,60 | 14 | 0,0001 | -4,98 | -2,22 | ICMS_post S1 IG INT |
| Les5_group | 0,28 | 0,80 | | 0,35 | 14 | 0,7351 | -1,44 | 2,00 | ICMS_post S1 IG INT |
| Les6_group | 2,64 | 0,80 | | 3,29 | 14 | **0,0053** | 0,92 | 4,36 | ICMS_post S1 IG INT |
| (Intercept) | -2,43 | 0,83 | | -2,94 | 14 | 1,07E-02 | -4,21 | -0,66 | ICMS_post iS1 SG INT |
| Les5_group | -1,86 | 1,03 | | -1,80 | 14 | 0,0937 | -4,07 | 0,36 | ICMS_post iS1 SG INT |
| Les6_group | 1,44 | 1,03 | | 1,40 | 14 | **0,1835** | -0,77 | 3,66 | ICMS_post iS1 SG INT |
| (Intercept) | -3,31 | 0,99 | | -3,33 | 14 | 0,0049 | -5,43 | -1,18 | ICMS_post iS1 G INT |
| Les5_group | -1,56 | 1,24 | | -1,26 | 14 | 0,2279 | -4,21 | 1,09 | ICMS_post iS1 G INT |
| Les6_group | 2,51 | 1,24 | | 2,03 | 14 | 0,0617 | -0,14 | 5,16 | ICMS_post iS1 G INT |
| (Intercept) | -6,10 | 1,80 | | -3,38 | 14 | 0,0045 | -9,96 | -2,23 | ICMS_post iS1 IG INT |
| Les5_group | -1,47 | 2,25 | | -0,65 | 14 | 0,5237 | -6,29 | 3,35 | ICMS_post iS1 IG INT |
| Les6_group | 3,54 | 2,25 | | 1,57 | 14 | 0,1379 | -1,28 | 8,36 | ICMS_post iS1 IG INT |

**Suppl. Table 2c: Statistical analysis of layer specific lesion effects using a liner mixed-effects model with LesV (threshold 5%) and LesVI (threshold 20%) as categorical variables (yes,no); pre and post muscimol condition separately**

| **Predictor** | **Estimate** | **SE** | **tStat** | **DF** | **pValue** | **Lower** | **Upper** | **Dependent Variable** |
| --- | --- | --- | --- | --- | --- | --- | --- | --- |
| (Intercept) | 36,84 | 5,84 | 6,31 | 14 | 0,0000 | 24,32 | 49,36 | AcS_pre AVR BF INT |
| Les5_group | -1,44 | 9,53 | -0,15 | 14 | 0,8822 | -21,88 | 19,00 | AcS_pre AVR BF INT |
| Les6_group | -9,39 | 9,40 | -1,00 | 14 | 0,3346 | -29,55 | 10,76 | AcS_pre AVR BF INT |
| (Intercept) | 16,52 | 0,48 | 34,12 | 12 | 2,55E-13 | 15,47 | 17,58 | AcS_pre AVR BF OL |
| Les5_group | 0,34 | 0,76 | 0,45 | 12 | 0,6609 | -1,31 | 1,99 | AcS_pre AVR BF OL |
| Les6_group | 0,76 | 0,74 | 1,03 | 12 | 0,3231 | -0,85 | 2,38 | AcS_pre AVR BF OL |
| (Intercept) | -66,45 | 11,88 | -5,60 | 14 | 0,0001 | -91,92 | -40,98 | AcS_pre S1 BF INT |
| Les5_group | -17,16 | 19,39 | -0,88 | 14 | 0,3913 | -58,75 | 24,44 | AcS_pre S1 BF INT |
| Les6_group | 27,22 | 19,12 | 1,42 | 14 | 0,1765 | -13,79 | 68,23 | AcS_pre S1 BF INT |
| (Intercept) | 18,97 | 0,46 | 40,88 | 14 | 5,74E-16 | 17,98 | 19,97 | AcS_pre S1 BF OL |
| Les5_group | 3,23 | 0,76 | 4,26 | 14 | **0,0008** | 1,61 | 4,86 | AcS_pre S1 BF OL |
| Les6_group | -1,57 | 0,75 | -2,10 | 14 | 0,0542 | -3,17 | 0,03 | AcS_pre S1 BF OL |
| (Intercept) | -13,91 | 3,75 | -3,71 | 14 | 0,0023 | -21,95 | -5,86 | AcS_pre S1 nBF INT |
| Les5_group | -1,56 | 6,13 | -0,25 | 14 | 0,8026 | -14,70 | 11,58 | AcS_pre S1 nBF INT |
| Les6_group | 1,78 | 6,04 | 0,29 | 14 | 0,7727 | -11,17 | 14,73 | AcS_pre S1 nBF INT |
| (Intercept) | 25,99 | 0,96 | 27,10 | 14 | 1,69E-13 | 23,93 | 28,04 | AcS_pre S1 nBF OL |
| Les5_group | 3,62 | 1,57 | 2,31 | 14 | **0,0367** | 0,26 | 6,97 | AcS_pre S1 nBF OL |
| Les6_group | -4,45 | 1,54 | -2,88 | 14 | **0,0120** | -7,76 | -1,14 | AcS_pre S1 nBF OL |
| (Intercept) | -80,77 | 3,59 | -22,52 | 14 | 2,14E-12 | -88,46 | -73,07 | AcS_pre S1 rel_nBF INT |
| Les5_group | -7,95 | 5,86 | -1,36 | 14 | 0,1964 | -20,51 | 4,62 | AcS_pre S1 rel_nBF INT |
| Les6_group | 15,74 | 5,78 | 2,73 | 14 | **0,0164** | 3,36 | 28,13 | AcS_pre S1 rel_nBF INT |
| (Intercept) | 7,01 | 0,87 | 8,04 | 14 | 1,29E-06 | 5,14 | 8,89 | AcS_pre S1 rel_nBF OL |
| Les5_group | 0,38 | 1,42 | 0,27 | 14 | 0,7914 | -2,67 | 3,44 | AcS_pre S1 rel_nBF OL |
| Les6_group | -2,88 | 1,40 | -2,05 | 14 | 0,0594 | -5,89 | 0,13 | AcS_pre S1 rel_nBF OL |
| (Intercept) | -19,33 | 3,26 | -5,93 | 14 | 0,0000 | -26,33 | -12,33 | AcS_pre iS1 BF INT |
| Les5_group | 2,22 | 5,33 | 0,42 | 14 | 0,6835 | -9,21 | 13,64 | AcS_pre iS1 BF INT |
| Les6_group | 12,02 | 5,25 | 2,29 | 14 | **0,0382** | 0,75 | 23,28 | AcS_pre iS1 BF INT |
| (Intercept) | 17,59 | 1,59 | 11,09 | 12 | 1,16E-07 | 14,13 | 21,05 | AcS_pre iS1 BF OL |
| Les5_group | -2,12 | 2,48 | -0,86 | 12 | 0,4091 | -7,51 | 3,28 | AcS_pre iS1 BF OL |
| Les6_group | 3,24 | 2,43 | 1,33 | 12 | 0,2080 | -2,06 | 8,53 | AcS_pre iS1 BF OL |
| (Intercept) | -6,16 | 1,62 | -3,80 | 14 | 0,0020 | -9,64 | -2,68 | AcS_pre iS1 nBF INT |
| Les5_group | 1,26 | 2,65 | 0,48 | 14 | 0,6408 | -4,42 | 6,95 | AcS_pre iS1 nBF INT |
| Les6_group | 0,65 | 2,61 | 0,25 | 14 | 0,8083 | -4,96 | 6,25 | AcS_pre iS1 nBF INT |
| (Intercept) | 21,89 | 2,20 | 9,94 | 13 | 1,92E-07 | 17,13 | 26,64 | AcS_pre iS1 nBF OL |
| Les5_group | -4,61 | 3,67 | -1,26 | 13 | 0,2311 | -12,55 | 3,32 | AcS_pre iS1 nBF OL |
| Les6_group | 4,87 | 3,56 | 1,37 | 13 | 0,1940 | -2,81 | 12,55 | AcS_pre iS1 nBF OL |
| (Intercept) | -72,32 | 24,81 | -2,92 | 13 | 0,0121 | -125,92 | -18,72 | AcS_pre S2 BF INT |
| Les5_group | 27,25 | 41,39 | 0,66 | 13 | 0,5217 | -62,16 | 116,66 | AcS_pre S2 BF INT |
| Les6_group | -56,90 | 40,07 | -1,42 | 13 | 0,1791 | -143,47 | 29,66 | AcS_pre S2 BF INT |
| (Intercept) | 20,09 | 1,67 | 12,02 | 11 | 1,14E-07 | 16,42 | 23,77 | AcS_pre S2 BF OL |
| Les5_group | -1,75 | 3,09 | -0,57 | 11 | 0,5821 | -8,57 | 5,06 | AcS_pre S2 BF OL |
| Les6_group | 1,08 | 3,00 | 0,36 | 11 | 0,7264 | -5,52 | 7,67 | AcS_pre S2 BF OL |
| (Intercept) | 8,99 | 40,95 | 0,22 | 13 | 0,8296 | -79,48 | 97,47 | AcS_pre S2 rel_BF INT |
| Les5_group | -82,33 | 68,32 | -1,21 | 13 | 0,2497 | -229,91 | 65,26 | AcS_pre S2 rel_BF INT |
| Les6_group | 224,45 | 66,15 | 3,39 | 13 | **0,0048** | 81,55 | 367,35 | AcS_pre S2 rel_BF INT |
| (Intercept) | -58,09 | 11,17 | -5,20 | 14 | 0,0001 | -82,05 | -34,13 | AcS_pre S3 BF INT |
| Les5_group | -21,06 | 18,24 | -1,15 | 14 | 0,2676 | -60,19 | 18,07 | AcS_pre S3 BF INT |
| Les6_group | 10,65 | 17,99 | 0,59 | 14 | 0,5631 | -27,93 | 49,23 | AcS_pre S3 BF INT |
| (Intercept) | 20,09 | 1,67 | 12,02 | 11 | 1,14E-07 | 16,42 | 23,77 | AcS_pre S3 BF OL |
| Les5_group | -1,75 | 3,09 | -0,57 | 11 | 0,5821 | -8,57 | 5,06 | AcS_pre S3 BF OL |
| Les6_group | 1,08 | 3,00 | 0,36 | 11 | 0,7264 | -5,52 | 7,67 | AcS_pre S3 BF OL |
| (Intercept) | -3,55 | 27,65 | -0,13 | 14 | 0,8997 | -62,85 | 55,75 | AcS_pre S3 rel_BF INT |
| Les5_group | 19,47 | 45,15 | 0,43 | 14 | 0,6729 | -77,38 | 116,31 | AcS_pre S3 rel_BF INT |
| Les6_group | 49,67 | 44,52 | 1,12 | 14 | 0,2833 | -45,82 | 145,16 | AcS_pre S3 rel_BF INT |
| (Intercept) | 8,66 | 1,81 | 4,77 | 13 | 0,0004 | 4,74 | 12,58 | AcS_post AVR BF INT |
| Les5_group | -2,24 | 3,03 | -0,74 | 13 | 0,4724 | -8,78 | 4,30 | AcS_post AVR BF INT |
| Les6_group | -1,17 | 2,93 | -0,40 | 13 | 0,6964 | -7,50 | 5,16 | AcS_post AVR BF INT |
| (Intercept) | 17,16 | 0,86 | 19,84 | 13 | 4,19E-11 | 15,29 | 19,03 | AcS_post AVR BF OL |
| Les5_group | 1,73 | 1,44 | 1,20 | 13 | 0,2525 | -1,39 | 4,84 | AcS_post AVR BF OL |
| Les6_group | -0,86 | 1,40 | -0,62 | 13 | 0,5470 | -3,88 | 2,15 | AcS_post AVR BF OL |
| (Intercept) | -16,25 | 3,92 | -4,15 | 13 | 0,0011 | -24,72 | -7,78 | AcS_post S1 BF INT |
| Les5_group | 1,82 | 6,54 | 0,28 | 13 | 0,7846 | -12,30 | 15,95 | AcS_post S1 BF INT |
| Les6_group | 5,12 | 6,33 | 0,81 | 13 | 0,4329 | -8,55 | 18,80 | AcS_post S1 BF INT |
| (Intercept) | 18,31 | 0,63 | 28,96 | 13 | 3,42E-13 | 16,94 | 19,67 | AcS_post S1 BF OL |
| Les5_group | 2,55 | 1,05 | 2,41 | 13 | **0,0313** | 0,27 | 4,82 | AcS_post S1 BF OL |
| Les6_group | -1,02 | 1,02 | -1,00 | 13 | 0,3348 | -3,23 | 1,18 | AcS_post S1 BF OL |
| (Intercept) | -3,35 | 1,64 | -2,04 | 12 | 0,0638 | -6,92 | 0,23 | AcS_post S1 nBF INT |
| Les5_group | -0,22 | 3,17 | -0,07 | 12 | 0,9456 | -7,13 | 6,69 | AcS_post S1 nBF INT |
| Les6_group | 0,05 | 3,00 | 0,02 | 12 | 0,9872 | -6,48 | 6,58 | AcS_post S1 nBF INT |
| (Intercept) | 23,00 | 1,36 | 16,88 | 9 | 4,03E-08 | 19,92 | 26,08 | AcS_post S1 nBF OL |
| Les5_group | 2,33 | 2,73 | 0,86 | 9 | 0,4141 | -3,83 | 8,50 | AcS_post S1 nBF OL |
| Les6_group | 1,17 | 2,36 | 0,49 | 9 | 0,6329 | -4,17 | 6,51 | AcS_post S1 nBF OL |
| (Intercept) | -81,31 | 4,75 | -17,11 | 12 | 8,53E-10 | -91,66 | -70,96 | AcS_post S1 rel_nBF INT |
| Les5_group | -7,09 | 9,18 | -0,77 | 12 | 0,4546 | -27,10 | 12,91 | AcS_post S1 rel_nBF INT |
| Les6_group | 15,56 | 8,67 | 1,79 | 12 | 0,0981 | -3,34 | 34,46 | AcS_post S1 rel_nBF INT |
| (Intercept) | 5,67 | 1,17 | 4,84 | 9 | 0,0009 | 3,02 | 8,31 | AcS_post S1 rel_nBF OL |
| Les5_group | 2,00 | 2,34 | 0,85 | 9 | 0,4151 | -3,30 | 7,30 | AcS_post S1 rel_nBF OL |
| Les6_group | 0,50 | 2,03 | 0,25 | 9 | 0,8108 | -4,09 | 5,09 | AcS_post S1 rel_nBF OL |
| (Intercept) | -75,37 | 3,31 | -22,76 | 13 | 7,40E-12 | -82,53 | -68,22 | AcS_post S1 rel_preBF INT |
| Les5_group | -10,16 | 5,53 | -1,84 | 13 | 0,0889 | -22,10 | 1,78 | AcS_post S1 rel_preBF INT |
| Les6_group | -2,32 | 5,35 | -0,43 | 13 | 0,6715 | -13,88 | 9,24 | AcS_post S1 rel_preBF INT |
| (Intercept) | -5,51 | 1,67 | -3,31 | 13 | 0,0057 | -9,11 | -1,91 | AcS_post iS1 BF INT |
| Les5_group | 1,04 | 2,78 | 0,37 | 13 | 0,7140 | -4,96 | 7,04 | AcS_post iS1 BF INT |
| Les6_group | 3,10 | 2,69 | 1,15 | 13 | 0,2705 | -2,72 | 8,91 | AcS_post iS1 BF INT |
| (Intercept) | 17,74 | 3,33 | 5,33 | 10 | 0,0003 | 10,32 | 25,15 | AcS_post iS1 BF OL |
| Les5_group | -6,17 | 5,76 | -1,07 | 10 | 0,3093 | -19,02 | 6,67 | AcS_post iS1 BF OL |
| Les6_group | 10,54 | 5,63 | 1,87 | 10 | 0,0904 | -1,99 | 23,08 | AcS_post iS1 BF OL |
| (Intercept) | -1,42 | 0,60 | -2,36 | 12 | 0,0362 | -2,74 | -0,11 | AcS_post iS1 nBF INT |
| Les5_group | 0,03 | 1,17 | 0,03 | 12 | 0,9766 | -2,51 | 2,58 | AcS_post iS1 nBF INT |
| Les6_group | 0,60 | 1,10 | 0,54 | 12 | 0,5971 | -1,80 | 3,00 | AcS_post iS1 nBF INT |
| (Intercept) | 10,27 | 1,57 | 6,54 | 15 | 0,0000 | 6,92 | 13,61 | ICMS_pre AVR SG INT |
| Les5_group | 1,02 | 2,35 | 0,43 | 15 | 0,6724 | -4,00 | 6,03 | ICMS_pre AVR SG INT |
| Les6_group | 0,30 | 2,34 | 0,13 | 15 | 0,9004 | -4,69 | 5,29 | ICMS_pre AVR SG INT |
| (Intercept) | 17,98 | 2,89 | 6,23 | 15 | 1,61E-05 | 11,83 | 24,13 | ICMS_pre AVR G INT |
| Les5_group | 1,84 | 4,33 | 0,43 | 15 | 0,6761 | -7,38 | 11,07 | ICMS_pre AVR G INT |
| Les6_group | -7,57 | 4,30 | -1,76 | 15 | 0,0988 | -16,74 | 1,60 | ICMS_pre AVR G INT |
| (Intercept) | 27,02 | 2,51 | 10,74 | 15 | 1,93E-08 | 21,66 | 32,38 | ICMS_pre AVR IG INT |
| Les5_group | 2,63 | 3,77 | 0,70 | 15 | 0,4961 | -5,41 | 10,67 | ICMS_pre AVR IG INT |
| Les6_group | -15,90 | 3,75 | -4,24 | 15 | **0,0007** | -23,89 | -7,90 | ICMS_pre AVR IG INT |
| (Intercept) | -2,00 | 0,48 | -4,13 | 15 | 0,0009 | -3,03 | -0,96 | ICMS_pre S1 SG INT |
| Les5_group | 0,45 | 0,73 | 0,62 | 15 | 0,5477 | -1,10 | 1,99 | ICMS_pre S1 SG INT |
| Les6_group | 1,47 | 0,72 | 2,04 | 15 | 0,0599 | -0,07 | 3,00 | ICMS_pre S1 SG INT |
| (Intercept) | -7,87 | 2,22 | -3,55 | 15 | 0,0029 | -12,59 | -3,14 | ICMS_pre S1 G INT |
| Les5_group | -2,78 | 3,33 | -0,83 | 15 | 0,4173 | -9,87 | 4,32 | ICMS_pre S1 G INT |
| Les6_group | 5,83 | 3,31 | 1,76 | 15 | 0,0981 | -1,22 | 12,88 | ICMS_pre S1 G INT |
| (Intercept) | -21,59 | 4,31 | -5,01 | 15 | 0,0002 | -30,77 | -12,40 | ICMS_pre S1 IG INT |
| Les5_group | -4,88 | 6,46 | -0,75 | 15 | 0,4620 | -18,66 | 8,90 | ICMS_pre S1 IG INT |
| Les6_group | 21,58 | 6,42 | 3,36 | 15 | **0,0043** | 7,89 | 35,27 | ICMS_pre S1 IG INT |
| (Intercept) | -3,12 | 0,77 | -4,04 | 15 | 1,08E-03 | -4,77 | -1,47 | ICMS_pre iS1 SG INT |
| Les5_group | 0,92 | 1,16 | 0,79 | 15 | 0,4403 | -1,55 | 3,39 | ICMS_pre iS1 SG INT |
| Les6_group | 2,05 | 1,15 | 1,78 | 15 | 0,0952 | -0,40 | 4,51 | ICMS_pre iS1 SG INT |
| (Intercept) | -4,36 | 0,90 | -4,83 | 15 | 0,0002 | -6,28 | -2,44 | ICMS_pre iS1 G INT |
| Les5_group | 1,51 | 1,35 | 1,12 | 15 | 0,2817 | -1,37 | 4,40 | ICMS_pre iS1 G INT |
| Les6_group | 2,75 | 1,35 | 2,04 | 15 | 0,0590 | -0,12 | 5,62 | ICMS_pre iS1 G INT |
| (Intercept) | -14,12 | 3,43 | -4,11 | 15 | 0,0009 | -21,43 | -6,80 | ICMS_pre iS1 IG INT |
| Les5_group | 4,10 | 5,15 | 0,80 | 15 | 0,4388 | -6,88 | 15,07 | ICMS_pre iS1 IG INT |
| Les6_group | 7,49 | 5,12 | 1,46 | 15 | 0,1638 | -3,42 | 18,40 | ICMS_pre iS1 IG INT |
| (Intercept) | 6,81 | 1,10 | 6,18 | 14 | 0,0000 | 4,44 | 9,17 | ICMS_post AVR SG INT |
| Les5_group | 7,30 | 1,68 | 4,35 | 14 | **0,0007** | 3,70 | 10,91 | ICMS_post AVR SG INT |
| Les6_group | 0,83 | 1,66 | 0,50 | 14 | 0,6240 | -2,72 | 4,38 | ICMS_post AVR SG INT |
| (Intercept) | 7,20 | 0,97 | 7,40 | 14 | 0,0000 | 5,12 | 9,29 | ICMS_post AVR G INT |
| Les5_group | 8,18 | 1,48 | 5,51 | 14 | **0,0001** | 5,00 | 11,36 | ICMS_post AVR G INT |
| Les6_group | -0,42 | 1,46 | -0,29 | 14 | 0,7773 | -3,56 | 2,72 | ICMS_post AVR G INT |
| (Intercept) | 8,34 | 1,08 | 7,74 | 14 | 1,99E-06 | 6,03 | 10,66 | ICMS_post AVR IG INT |
| Les5_group | 9,25 | 1,64 | 5,63 | 14 | **0,0001** | 5,72 | 12,77 | ICMS_post AVR IG INT |
| Les6_group | -2,64 | 1,62 | -1,63 | 14 | 0,1259 | -6,11 | 0,84 | ICMS_post AVR IG INT |
| (Intercept) | -1,08 | 0,35 | -3,06 | 14 | 8,40E-03 | -1,83 | -0,32 | ICMS_post S1 SG INT |
| Les5_group | -0,23 | 0,54 | -0,43 | 14 | 0,6722 | -1,38 | 0,92 | ICMS_post S1 SG INT |
| Les6_group | 1,17 | 0,53 | 2,22 | 14 | **0,0436** | 0,04 | 2,31 | ICMS_post S1 SG INT |
| (Intercept) | -2,28 | 0,58 | -3,93 | 14 | 0,0015 | -3,52 | -1,04 | ICMS_post S1 G INT |
| Les5_group | -0,78 | 0,88 | -0,88 | 14 | 0,3920 | -2,67 | 1,11 | ICMS_post S1 G INT |
| Les6_group | 2,33 | 0,87 | 2,68 | 14 | **0,0179** | 0,47 | 4,20 | ICMS_post S1 G INT |
| (Intercept) | -3,29 | 0,47 | -6,94 | 14 | 0,0000 | -4,30 | -2,27 | ICMS_post S1 IG INT |
| Les5_group | -0,55 | 0,72 | -0,76 | 14 | 0,4573 | -2,10 | 1,00 | ICMS_post S1 IG INT |
| Les6_group | 3,37 | 0,71 | 4,73 | 14 | **0,0003** | 1,84 | 4,89 | ICMS_post S1 IG INT |
| (Intercept) | -2,14 | 0,73 | -2,94 | 14 | 1,08E-02 | -3,71 | -0,58 | ICMS_post iS1 SG INT |
| Les5_group | -2,14 | 1,11 | -1,93 | 14 | 0,0748 | -4,52 | 0,24 | ICMS_post iS1 SG INT |
| Les6_group | 1,43 | 1,10 | 1,31 | 14 | 0,2121 | -0,92 | 3,78 | ICMS_post iS1 SG INT |
| (Intercept) | -2,73 | 0,90 | -3,05 | 14 | 0,0086 | -4,65 | -0,81 | ICMS_post iS1 G INT |
| Les5_group | -1,96 | 1,36 | -1,44 | 14 | 0,1721 | -4,89 | 0,96 | ICMS_post iS1 G INT |
| Les6_group | 2,28 | 1,35 | 1,69 | 14 | 0,1130 | -0,61 | 5,16 | ICMS_post iS1 G INT |
| (Intercept) | -5,02 | 1,65 | -3,05 | 14 | 0,0087 | -8,55 | -1,49 | ICMS_post iS1 IG INT |
| Les5_group | -1,67 | 2,51 | -0,67 | 14 | 0,5164 | -7,05 | 3,71 | ICMS_post iS1 IG INT |
| Les6_group | 2,32 | 2,48 | 0,94 | 14 | 0,3654 | -2,99 | 7,62 | ICMS_post iS1 IG INT |

**Suppl. Table 3: Linear mixed-effects model analysis (two-way mixed-design) to assess muscimol treatment effects using individual percentage cell loss in layers V and VI**

| **Predictor** | **Estimate** | **SE** | **tStat** | **DF** | **pValue** | **Lower** | **Upper** | **Dependent Variable** |
| --- | --- | --- | --- | --- | --- | --- | --- | --- |
| (Intercept) | 38,33 | 5,21 | 7,35 | 27 | 6,56E-08 | 27,64 | 49,03 | AcS AVR BF INT |
| LesV | -0,06 | 0,51 | -0,12 | 27 | 0,9044 | -1,11 | 0,98 | AcS AVR BF INT |
| LesVI | -0,34 | 0,24 | -1,43 | 27 | 0,1635 | -0,83 | 0,15 | AcS AVR BF INT |
| Treatment_pos | -29,98 | 5,62 | -5,34 | 27 | **1,23E-05** | -41,50 | -18,46 | AcS AVR BF INT |
| LesV:Treatment_pos | -0,06 | 0,55 | -0,12 | 27 | 0,9082 | -1,19 | 1,06 | AcS AVR BF INT |
| LesVI:Treatment_pos | 0,30 | 0,26 | 1,15 | 27 | 0,2609 | -0,24 | 0,84 | AcS AVR BF INT |
| (Intercept) | 16,36 | 0,81 | 20,17 | 25 | 5,55E-17 | 14,69 | 18,03 | AcS AVR BF OL |
| LesV | 0,04 | 0,08 | 0,45 | 25 | 0,6546 | -0,13 | 0,21 | AcS AVR BF OL |
| LesVI | 0,03 | 0,04 | 0,90 | 25 | 0,3776 | -0,04 | 0,11 | AcS AVR BF OL |
| Treatment_pos | 0,70 | 0,95 | 0,73 | 25 | 0,4716 | -1,27 | 2,66 | AcS AVR BF OL |
| LesV:Treatment_pos | 0,11 | 0,10 | 1,15 | 25 | 0,2619 | -0,09 | 0,31 | AcS AVR BF OL |
| LesVI:Treatment_pos | -0,04 | 0,04 | -0,94 | 25 | 0,3563 | -0,13 | 0,05 | AcS AVR BF OL |
| (Intercept) | -78,04 | 10,24 | -7,62 | 27 | 3,39E-08 | -99,05 | -57,02 | AcS S1 BF INT |
| LesV | -1,24 | 1,00 | -1,24 | 27 | 0,2270 | -3,29 | 0,82 | AcS S1 BF INT |
| LesVI | 1,17 | 0,47 | 2,50 | 27 | **0,0188** | 0,21 | 2,13 | AcS S1 BF INT |
| Treatment_pos | 60,24 | 10,29 | 5,85 | 27 | **3,12E-06** | 39,13 | 81,36 | AcS S1 BF INT |
| LesV:Treatment_pos | 1,33 | 1,00 | 1,33 | 27 | 0,1959 | -0,73 | 3,39 | AcS S1 BF INT |
| LesVI:Treatment_pos | -0,91 | 0,48 | -1,89 | 27 | 0,0691 | -1,89 | 0,08 | AcS S1 BF INT |
| (Intercept) | 19,58 | 0,69 | 28,51 | 27 | 1,09E-21 | 18,17 | 20,99 | AcS S1 BF OL |
| **LesV** | 0,21 | 0,07 | 3,11 | 27 | **0,0043** | 0,07 | 0,35 | AcS S1 BF OL |
| LesVI | -0,04 | 0,03 | -1,38 | 27 | 0,1788 | -0,11 | 0,02 | AcS S1 BF OL |
| Treatment_pos | -1,00 | 0,67 | -1,50 | 27 | 0,1442 | -2,37 | 0,37 | AcS S1 BF OL |
| LesV:Treatment_pos | -0,05 | 0,07 | -0,70 | 27 | 0,4927 | -0,18 | 0,09 | AcS S1 BF OL |
| LesVI:Treatment_pos | 0,03 | 0,03 | 0,81 | 27 | 0,4275 | -0,04 | 0,09 | AcS S1 BF OL |
| (Intercept) | -13,54 | 3,47 | -3,90 | 26 | 0,0006 | -20,67 | -6,41 | AcS S1 nBF INT |
| LesV | -0,17 | 0,34 | -0,51 | 26 | 0,6159 | -0,87 | 0,52 | AcS S1 nBF INT |
| LesVI | 0,03 | 0,16 | 0,18 | 26 | 0,8622 | -0,30 | 0,35 | AcS S1 nBF INT |
| Treatment_pos | 9,09 | 4,69 | 1,94 | 26 | 0,0633 | -0,54 | 18,73 | AcS S1 nBF INT |
| LesV:Treatment_pos | 0,01 | 0,50 | 0,02 | 26 | 0,9847 | -1,02 | 1,04 | AcS S1 nBF INT |
| LesVI:Treatment_pos | 0,05 | 0,22 | 0,22 | 26 | 0,8299 | -0,41 | 0,51 | AcS S1 nBF INT |
| (Intercept) | 27,90 | 1,19 | 23,41 | 23 | 1,52E-17 | 25,44 | 30,37 | AcS S1 nBF OL |
| LesV | 0,26 | 0,12 | 2,24 | 23 | **0,0350** | 0,02 | 0,50 | AcS S1 nBF OL |
| LesVI | -0,19 | 0,05 | -3,42 | 23 | **0,0023** | -0,30 | -0,07 | AcS S1 nBF OL |
| Treatment_pos | -5,15 | 1,54 | -3,34 | 23 | **0,0028** | -8,35 | -1,96 | AcS S1 nBF OL |
| LesV:Treatment_pos | -0,16 | 0,19 | -0,85 | 23 | 0,4053 | -0,54 | 0,23 | AcS S1 nBF OL |
| LesVI:Treatment_pos | 0,25 | 0,07 | 3,53 | 23 | **0,0018** | 0,10 | 0,40 | AcS S1 nBF OL |
| (Intercept) | -87,47 | 4,72 | -18,55 | 26 | 1,63E-16 | -97,16 | -77,77 | AcS S1 rel_nBF INT |
| LesV | -0,40 | 0,46 | -0,87 | 26 | 0,3920 | -1,35 | 0,55 | AcS S1 rel_nBF INT |
| LesVI | 0,66 | 0,21 | 3,06 | 26 | **0,0050** | 0,22 | 1,10 | AcS S1 rel_nBF INT |
| Treatment_pos | 7,02 | 7,05 | 1,00 | 26 | 0,3288 | -7,48 | 21,51 | AcS S1 rel_nBF INT |
| LesV:Treatment_pos | 0,86 | 0,74 | 1,15 | 26 | 0,2587 | -0,67 | 2,38 | AcS S1 rel_nBF INT |
| LesVI:Treatment_pos | -0,45 | 0,33 | -1,35 | 26 | 0,1884 | -1,14 | 0,23 | AcS S1 rel_nBF INT |
| (Intercept) | 8,32 | 1,05 | 7,91 | 23 | 5,23E-08 | 6,15 | 10,50 | AcS S1 rel_nBF OL |
| LesV | 0,05 | 0,10 | 0,51 | 23 | 0,6177 | -0,16 | 0,26 | AcS S1 rel_nBF OL |
| LesVI | -0,14 | 0,05 | -2,97 | 23 | **0,0068** | -0,24 | -0,04 | AcS S1 rel_nBF OL |
| Treatment_pos | -2,70 | 1,47 | -1,84 | 23 | 0,0788 | -5,74 | 0,34 | AcS S1 rel_nBF OL |
| LesV:Treatment_pos | 0,04 | 0,18 | 0,22 | 23 | 0,8290 | -0,33 | 0,40 | AcS S1 rel_nBF OL |
| LesVI:Treatment_pos | 0,17 | 0,07 | 2,56 | 23 | **0,0176** | 0,03 | 0,32 | AcS S1 rel_nBF OL |
| (Intercept) | -21,64 | 3,08 | -7,03 | 27 | 1,47E-07 | -27,95 | -15,32 | AcS iS1 BF INT |
| LesV | 0,14 | 0,30 | 0,45 | 27 | 0,6545 | -0,48 | 0,75 | AcS iS1 BF INT |
| LesVI | 0,45 | 0,14 | 3,23 | 27 | **0,0033** | 0,16 | 0,74 | AcS iS1 BF INT |
| Treatment_pos | 15,17 | 3,02 | 5,02 | 27 | **2,88E-05** | 8,97 | 21,37 | AcS iS1 BF INT |
| LesV:Treatment_pos | -0,12 | 0,29 | -0,39 | 27 | 0,6991 | -0,72 | 0,49 | AcS iS1 BF INT |
| LesVI:Treatment_pos | -0,31 | 0,14 | -2,18 | 27 | **0,0380** | -0,60 | -0,02 | AcS iS1 BF INT |
| (Intercept) | 15,93 | 2,78 | 5,73 | 22 | 9,20E-06 | 10,17 | 21,70 | AcS iS1 BF OL |
| LesV | -0,03 | 0,30 | -0,10 | 22 | 0,9187 | -0,64 | 0,58 | AcS iS1 BF OL |
| LesVI | 0,12 | 0,13 | 0,97 | 22 | 0,3411 | -0,14 | 0,38 | AcS iS1 BF OL |
| Treatment_pos | -1,03 | 2,78 | -0,37 | 22 | 0,7145 | -6,80 | 4,74 | AcS iS1 BF OL |
| LesV:Treatment_pos | -0,08 | 0,31 | -0,25 | 22 | 0,8018 | -0,72 | 0,56 | AcS iS1 BF OL |
| LesVI:Treatment_pos | 0,21 | 0,13 | 1,54 | 22 | 0,1371 | -0,07 | 0,48 | AcS iS1 BF OL |
| (Intercept) | -5,98 | 1,48 | -4,03 | 26 | 0,0004 | -9,03 | -2,93 | AcS iS1 nBF INT |
| LesV | 0,08 | 0,14 | 0,54 | 26 | 0,5921 | -0,22 | 0,38 | AcS iS1 nBF INT |
| LesVI | 0,02 | 0,07 | 0,29 | 26 | 0,7749 | -0,12 | 0,16 | AcS iS1 nBF INT |
| Treatment_pos | 4,11 | 2,05 | 2,01 | 26 | 0,0551 | -0,10 | 8,32 | AcS iS1 nBF INT |
| LesV:Treatment_pos | -0,12 | 0,22 | -0,55 | 26 | 0,5840 | -0,57 | 0,33 | AcS iS1 nBF INT |
| LesVI:Treatment_pos | 0,03 | 0,10 | 0,26 | 26 | 0,7932 | -0,17 | 0,23 | AcS iS1 nBF INT |
| (Intercept) | 10,55 | 1,65 | 6,41 | 29 | 5,23E-07 | 7,19 | 13,92 | ICMS AVR SG INT |
| LesV | 0,03 | 0,17 | 0,17 | 29 | 0,8685 | -0,31 | 0,37 | ICMS AVR SG INT |
| LesVI | 0,01 | 0,08 | 0,14 | 29 | 0,8878 | -0,14 | 0,17 | ICMS AVR SG INT |
| Treatment_pos | -1,83 | 0,95 | -1,92 | 29 | 0,0643 | -3,77 | 0,12 | ICMS AVR SG INT |
| LesV:Treatment_pos | 0,49 | 0,10 | 5,18 | 29 | **1,53E-05** | 0,30 | 0,69 | ICMS AVR SG INT |
| LesVI:Treatment_pos | -0,03 | 0,04 | -0,76 | 29 | 0,4540 | -0,13 | 0,06 | ICMS AVR SG INT |
| (Intercept) | 19,99 | 2,52 | 7,95 | 29 | 9,19E-09 | 14,85 | 25,14 | ICMS AVR G INT |
| LesV | -0,29 | 0,25 | -1,16 | 29 | 0,2559 | -0,81 | 0,22 | ICMS AVR G INT |
| LesVI | -0,20 | 0,12 | -1,73 | 29 | 0,0934 | -0,44 | 0,04 | ICMS AVR G INT |
| Treatment_pos | -10,41 | 2,46 | -4,23 | 29 | **0,0002** | -15,44 | -5,38 | ICMS AVR G INT |
| LesV:Treatment_pos | 0,85 | 0,25 | 3,43 | 29 | **0,0018** | 0,34 | 1,35 | ICMS AVR G INT |
| LesVI:Treatment_pos | 0,14 | 0,12 | 1,21 | 29 | 0,2366 | -0,10 | 0,38 | ICMS AVR G INT |
| (Intercept) | 31,54 | 2,13 | 14,79 | 29 | 4,80E-15 | 27,18 | 35,90 | ICMS AVR IG INT |
| LesV | -0,15 | 0,21 | -0,70 | 29 | 0,4892 | -0,59 | 0,29 | ICMS AVR IG INT |
| LesVI | -0,56 | 0,10 | -5,69 | 29 | **3,78E-06** | -0,76 | -0,36 | ICMS AVR IG INT |
| Treatment_pos | -20,38 | 2,46 | -8,28 | 29 | **3,97E-09** | -25,41 | -15,34 | ICMS AVR IG INT |
| LesV:Treatment_pos | 0,79 | 0,25 | 3,21 | 29 | **0,0033** | 0,29 | 1,30 | ICMS AVR IG INT |
| LesVI:Treatment_pos | 0,43 | 0,12 | 3,74 | 29 | **0,0008** | 0,20 | 0,67 | ICMS AVR IG INT |
| (Intercept) | -1,93 | 0,51 | -3,77 | 29 | 0,0007 | -2,98 | -0,88 | ICMS S1 SG INT |
| LesV | 0,07 | 0,05 | 1,37 | 29 | 0,1817 | -0,03 | 0,18 | ICMS S1 SG INT |
| LesVI | 0,03 | 0,02 | 1,33 | 29 | 0,1951 | -0,02 | 0,08 | ICMS S1 SG INT |
| Treatment_pos | 0,81 | 0,33 | 2,46 | 29 | **0,0199** | 0,14 | 1,49 | ICMS S1 SG INT |
| LesV:Treatment_pos | -0,07 | 0,03 | -2,16 | 29 | **0,0388** | -0,14 | 0,00 | ICMS S1 SG INT |
| LesVI:Treatment_pos | -0,01 | 0,02 | -0,34 | 29 | 0,7336 | -0,04 | 0,03 | ICMS S1 SG INT |
| (Intercept) | -10,48 | 1,82 | -5,74 | 29 | 3,22E-06 | -14,21 | -6,75 | ICMS S1 G INT |
| LesV | 0,08 | 0,18 | 0,46 | 29 | 0,6491 | -0,29 | 0,46 | ICMS S1 G INT |
| LesVI | 0,21 | 0,08 | 2,46 | 29 | **0,0200** | 0,03 | 0,38 | ICMS S1 G INT |
| Treatment_pos | 7,70 | 2,12 | 3,64 | 29 | **0,0011** | 3,37 | 12,04 | ICMS S1 G INT |
| LesV:Treatment_pos | -0,08 | 0,21 | -0,37 | 29 | 0,7163 | -0,51 | 0,36 | ICMS S1 G INT |
| LesVI:Treatment_pos | -0,14 | 0,10 | -1,38 | 29 | 0,1789 | -0,34 | 0,07 | ICMS S1 G INT |
| (Intercept) | -28,10 | 3,33 | -8,43 | 29 | 0,0000 | -34,92 | -21,28 | ICMS S1 IG INT |
| LesV | -0,14 | 0,33 | -0,42 | 29 | 0,6746 | -0,83 | 0,54 | ICMS S1 IG INT |
| LesVI | 0,81 | 0,15 | 5,31 | 29 | **1,06E-05** | 0,50 | 1,13 | ICMS S1 IG INT |
| Treatment_pos | 24,27 | 4,45 | 5,45 | 29 | **7,28E-06** | 15,16 | 33,37 | ICMS S1 IG INT |
| LesV:Treatment_pos | 0,16 | 0,45 | 0,36 | 29 | 0,7188 | -0,75 | 1,08 | ICMS S1 IG INT |
| LesVI:Treatment_pos | -0,71 | 0,21 | -3,43 | 29 | **0,0018** | -1,14 | -0,29 | ICMS S1 IG INT |
| (Intercept) | -3,05 | 0,88 | -3,49 | 29 | 0,0016 | -4,84 | -1,26 | ICMS iS1 SG INT |
| LesV | 0,04 | 0,09 | 0,46 | 29 | 0,6503 | -0,14 | 0,22 | ICMS iS1 SG INT |
| LesVI | 0,06 | 0,04 | 1,56 | 29 | 0,1285 | -0,02 | 0,15 | ICMS iS1 SG INT |
| Treatment_pos | -0,13 | 1,12 | -0,12 | 29 | 0,9082 | -2,42 | 2,16 | ICMS iS1 SG INT |
| LesV:Treatment_pos | -0,21 | 0,11 | -1,87 | 29 | 0,0709 | -0,44 | 0,02 | ICMS iS1 SG INT |
| LesVI:Treatment_pos | 0,01 | 0,05 | 0,27 | 29 | 0,7915 | -0,09 | 0,12 | ICMS iS1 SG INT |
| (Intercept) | -4,31 | 1,03 | -4,21 | 29 | 0,0002 | -6,41 | -2,21 | ICMS iS1 G INT |
| LesV | 0,05 | 0,10 | 0,52 | 29 | 0,6072 | -0,16 | 0,26 | ICMS iS1 G INT |
| LesVI | 0,09 | 0,05 | 1,98 | 29 | 0,0575 | 0,00 | 0,19 | ICMS iS1 G INT |
| Treatment_pos | 0,23 | 1,17 | 0,19 | 29 | 0,8481 | -2,16 | 2,62 | ICMS iS1 G INT |
| LesV:Treatment_pos | -0,24 | 0,12 | -2,04 | 29 | 0,0502 | -0,48 | 0,00 | ICMS iS1 G INT |
| LesVI:Treatment_pos | 0,03 | 0,05 | 0,55 | 29 | 0,5870 | -0,08 | 0,14 | ICMS iS1 G INT |
| (Intercept) | -16,65 | 2,90 | -5,74 | 29 | 3,29E-06 | -22,59 | -10,71 | ICMS iS1 IG INT |
| LesV | 0,37 | 0,29 | 1,28 | 29 | 0,2118 | -0,22 | 0,97 | ICMS iS1 IG INT |
| LesVI | 0,35 | 0,13 | 2,60 | 29 | **0,0144** | 0,07 | 0,62 | ICMS iS1 IG INT |
| Treatment_pos | 9,51 | 3,04 | 3,13 | 29 | **0,0040** | 3,29 | 15,73 | ICMS iS1 IG INT |
| LesV:Treatment_pos | -0,51 | 0,30 | -1,67 | 29 | 0,1059 | -1,13 | 0,11 | ICMS iS1 IG INT |
| LesVI:Treatment_pos | -0,19 | 0,14 | -1,35 | 29 | 0,1873 | -0,49 | 0,10 | ICMS iS1 IG INT |
